# Exploiting Sn(ii)/Sn(iv) redox chemistry: a silyl-stannylene offering close-to-thermoneutral transfer hydrogenation

**DOI:** 10.1039/d6dt02289h

**Published:** 2026-10-05

**Authors:** Aidan J. Murray, Agamemnon E. Crumpton, Simon Aldridge

**Affiliations:** a Inorganic Chemistry Laboratory, Department of Chemistry, University of Oxford South Parks Road Oxford OX1 3QR UK simon.aldridge@chem.ox.ac.uk

## Abstract

The aryl(silyl)stannylene Ar^Mes^Sn{Si(SiMe_3_)_3_} (1, Ar^Mes^ = 2,6-Mes_2_C_6_H_3_, Mes = 2,4,6-Me_3_C_6_H_2_) featuring a strongly electron donating silyl substituent has been shown to offer a close to thermodynamically neutral platform for the shuttling of H_2_ (Δ*G*° = −3.7 kcal mol^−1^ for H_2_ uptake). While the direct activation and release of dihydrogen itself is precluded by prohibitively high kinetic barriers (Δ*G*^‡^ = −35.7 kcal mol^−1^), 1 will dehydrogenate both ammonia- and dimethylamine-borane to yield the tin(iv) dihydride (1)H_2_. (1)H_2_, in turn, is shown to be competent for hydrogen transfer to an NHC, yielding the corresponding dihydro-imidazole and regenerating the Sn(ii) state in the form of the stannylene-carbene adduct 1·IMe_4_.

## Abstract 2

The silyl-stannylene Ar^Mes^Sn{Si(SiMe_3_)_3_} (1) offers close-to-thermoneutral shuttling of H_2_ (Δ*G*° = −3.7 kcal mol^−1^ for uptake); 1 dehydrogenates amine-boranes to yield the tin(iv) dihydride (1)H_2_, which is competent for hydrogen transfer to an NHC.

## Graphical abstract

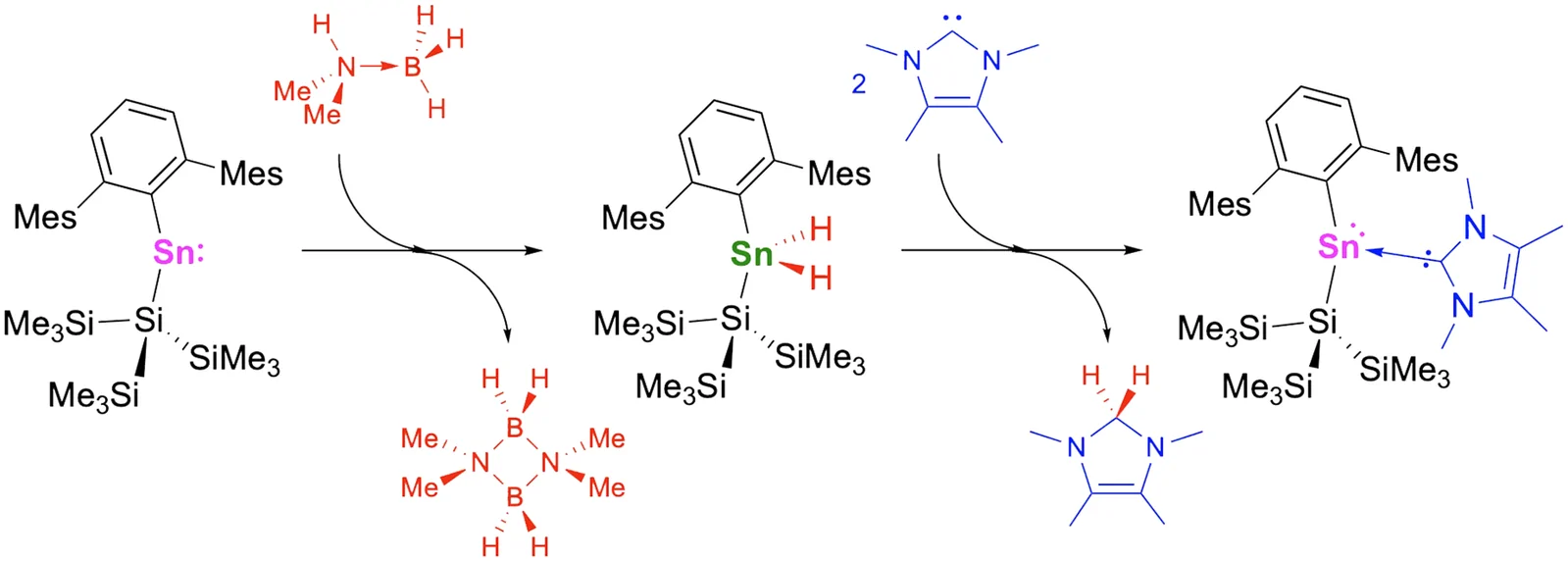

## Introduction

The activation of H_2_ is a key step in hydrogenation processes, and is accomplished primarily through the use of heavier late transition elements and their complexes.^1^ In attempting to move away from reliance on platinum group metals, a number of main group systems have been developed which activate hydrogen under mild conditions These include heavier group 14 alkyne analogues (RMMR; M = Ge, Sn), frustrated Lewis pairs (FLPs) and carbenes (CR_2_).^2–8^ The cleavage of H_2_ by carbenes (and their heavier group 14 metallylene analogues) involves synergistic interactions of the H_2_ σ and σ* orbitals with the LUMO and HOMO, respectively, of the tetrylene.^9^ The magnitude of the HOMO–LUMO energy separation is therefore critical to such reactivity, and has been shown to be tuneable through variation of the R-substituents.^8^ Thus, for example, Bertrand *et al.* showed that alkyl(amido)carbenes will oxidatively cleave H_2_ at the carbenic centre (Scheme 1), while under similar conditions bis(amido)carbenes remain inert, due to the wider HOMO–LUMO gap associated with a second π-donor substituent in the α position.^10^

**Scheme 1:**
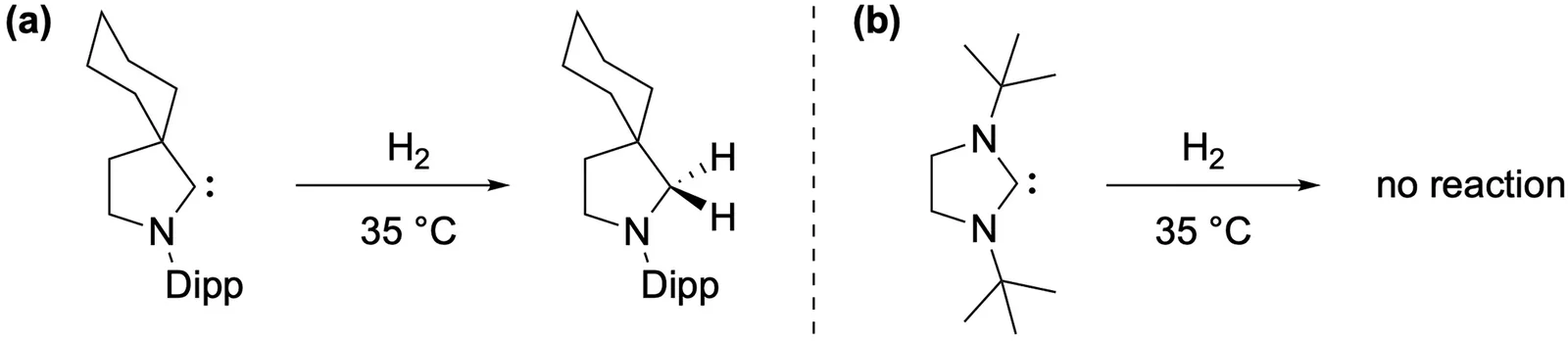
(a) Cleavage of dihydrogen by an alkyl(amido)carbene; (b) contrasting inertness of a bis(amido)carbene. (Dipp = 2,6-^i^Pr_2_C_6_H_3_).

In cases where the direct activation of H_2_ proves kinetically inaccessible, ammonia borane (H_3_N·BH_3_, AB) and related *N*-substituted amine boranes (RH_2_N·BH_3_ or R_2_HN·BH_3_) have been explored as dihydrogen ‘surrogates’, and many transition metal complexes have been shown to exploit these substrates as sources of H_2_.^11–16^ Recently, a number of main group systems have been shown to effect analogous stoichiometric (and even catalytic) ammonia- or amine-borane dehydrogenation, and in some cases bring about transfer hydrogenation of unsaturated substrates.^17,18^

In terms of developing redox-based catalytic cycles, tin has recently been shown to offer some promise,^19,20^ due to the accessibility of both Sn(ii) and Sn(iv) states in the presence of a suitable supporting ligand scaffold.^21,22^ Stannylenes (the tin analogues of carbenes, SnR_2_) are potential candidates for this purpose, showing evidence for both facile oxidative addition and reductive elimination processes in their reactivity towards certain small molecules. As with carbenes, the substituents employed at R have been shown to be highly influential in determining the HOMO–LUMO energy separation and hence reactivity profiles.^23–25^ The bulky, terphenyl-stabilised bis(aryl)stannylene (Ar^Dipp^)_2_Sn (Ar^Dipp^ = 2,6-Dipp_2_C_6_H_3_, Dipp = 2,6-^i^Pr_2_C_6_H_3_) introduced by Power *et al.*, has been shown to activate H_2_, although in this case arene elimination occurs, leading to the formation of a hydride-bridged Sn(ii) product (Scheme 2a).^26^ On the other hand, by employing more strongly electron-releasing boryl substituents, the bis(boryl)stannylene {(HCDippN)_2_B}_2_Sn could be shown to oxidatively add H_2_ to give the corresponding Sn(iv) dihydride (Scheme 2b).^27^ To our knowledge this represented the first example of a Sn(iv) dihydride derived from the insertion of a Sn(ii) species into the H–H bond of dihydrogen. On the other hand, Roesky and co-workers have reported the ligand-assisted hydrogenation of a germylene by AB (Scheme 2c), under conditions under which it is inert to H_2_ itself. Such reactivity implies that AB may serve as a suitable reagent for the (net) hydrogenation of group 14 species under mild conditions.^28^ Despite this, at the time of writing there have been very few reports detailing the activity of stannylenes toward ammonia borane,^19*b*^ and the only *amine* borane which has been shown to react (with {(HCDippN)_2_B}_2_Sn) is Me_3_N·BH_3_, which lacks the N–H bond(s) required to act as a H_2_ transfer agent (Scheme 2b).^27^

**Scheme 2:**
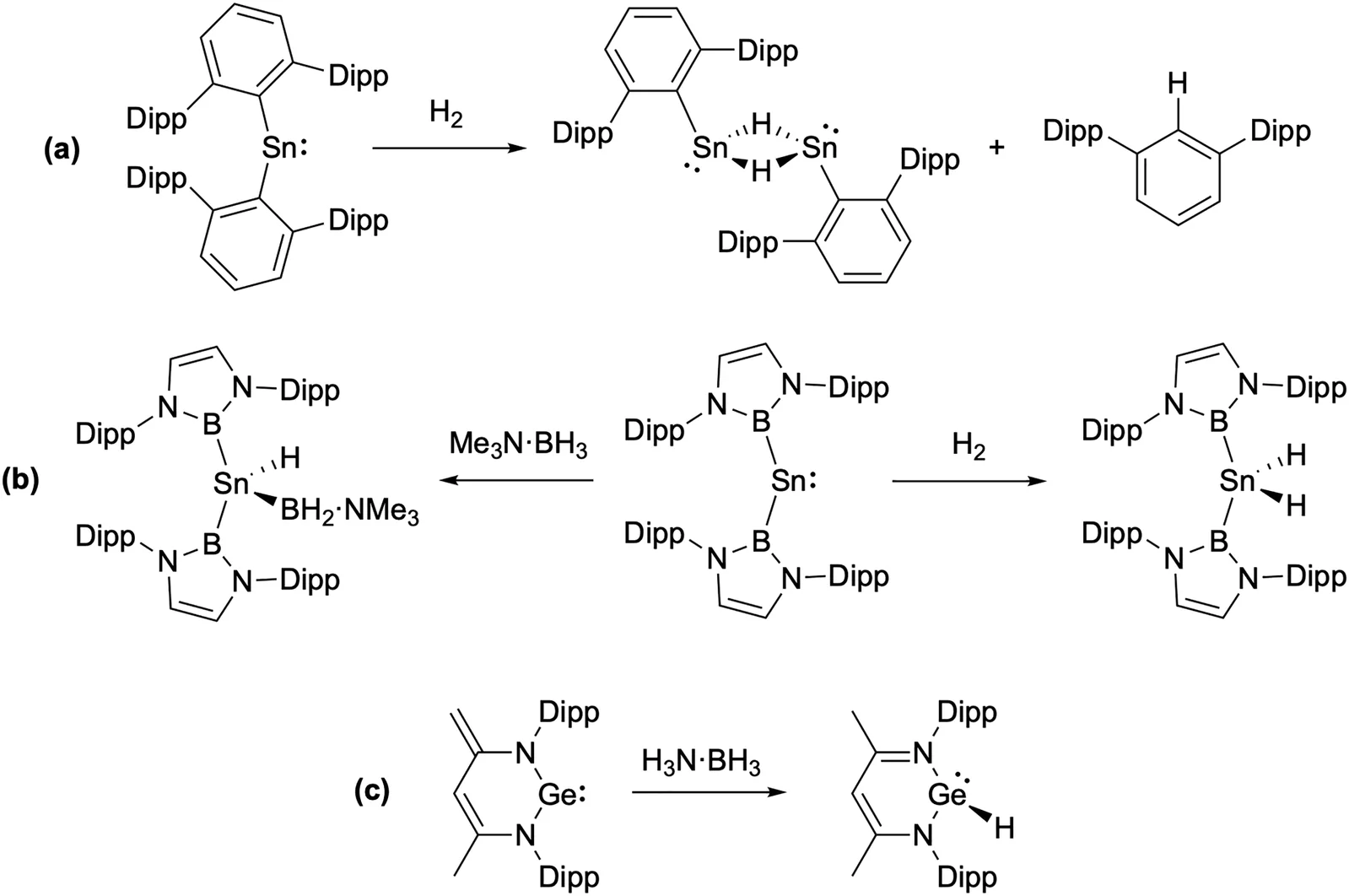
(a) Reaction of dihydrogen with (Ar^Dipp^)_2_Sn to give a dimeric Sn(ii) bridged hydride with arene elimination; (b) oxidative addition of dihydrogen to a bis(boryl)stannylene to produce an Sn(iv) dihydride; related B–H activation of Me_3_N·BH_3_; (c) ligand assisted reaction of a germylene with ammonia borane. (Ar^Dipp^ = 2,6-Dipp_2_C_6_H_3_).

The enhanced reactivity shown by boryl-ligated stannylenes prompted us to investigate another family of strongly electron-releasing substituents – namely silyl ligands – in order to promote access to the Sn(iv) state.^29^ Given the thermal fragility associated with bis(silyl)stannylenes, however,^32^ we focussed instead on the mixed aryl(silyl)stannylene Ar^Mes^Sn{Si(SiMe_3_)_3_} (1; Ar^Mes^ = 2,6-Mes_2_C_6_H_3_, Mes = 2,4,6-Me_3_C_6_H_2_, see Scheme 1), as a potential platform for the activation of H_2_ and sources thereof.^30–35^ In the event, we find that, while 1 can act as a vehicle for shuttling the elements of H_2_, such reactivity is restricted to indirect uptake/delivery on kinetic grounds.

## Results and discussion

### Reaction of 1 with sources of dihydrogen

We recently reported the synthesis of the homoleptic bis(silyl)stannylene {(Me_3_Si)_2_(^*t*^BuPh_2_Si)Si}_2_Sn, which is monomeric both in solution and in the solid state, but this compound does not react with H_2_ under ambient conditions, and decomposes to the disilane {(Me_3_Si)_2_(^*t*^BuPh_2_Si)Si}_2_ on gentle warming.^32^ Turning instead to the readily-synthesised aryl(silyl)stannylene Ar^Mes^Sn{Si(SiMe_3_)_3_} (1),^30^ we found that this system also shows no evidence for a direct reaction with H_2_, nor with 1,2-dihydronaphthalene or 9,10-dihydro-anthracene even at 80 °C over a period of several days. This scenario contrasts with the reactivity observed for the corresponding germylene, Ar^Mes^Ge{Si(SiMe_3_)_3_}, which cleaves H_2_ (at room temperature and 4 atm pressure) to give Ar^Mes^Ge(H)_2_{Si(SiMe_3_)_3_} in good yield over *ca.* 3 h.^27*b*^ This observation is consistent with the higher calculated HOMO–LUMO energy separation for 1 compared to its germylene counterpart (184 kJ mol^−1^*vs.* 134 kJ mol^−1^; Fig. S18).^27*b*^ On the other hand, combining 1 with the more polar, protic/hydridic reagent AB in C_6_D_6_ solution at 50 °C results in discharge of the green colour of the solution over *ca.* 12 h (Scheme 3). *In situ*^11^B{^1^H} NMR monitoring shows the formation of a mixture of products including borazine (B_3_N_3_H_6_; *δ*_B_ = 31 ppm), together with several aminoborane aggregates, [NH_2_BH_2_]_*n*_, giving rise to signals in the range *δ*_B_ = −6 to −24 ppm.^36,37^ The ^1^H NMR spectrum shows single sets of new Ar^Mes^ and Si(SiMe_3_)_3_ resonances, shifted from those of 1, in addition to a new singlet at *δ*_H_ = 4.23 ppm, integrating to two protons and possessing ^119/117^Sn satellites (^1^*J*_Sn–H_ = 1580, 1510 Hz).^30^ Further spectroscopic analysis of the (colourless) isolated product reveals a triplet in the ^119^Sn NMR spectrum centred at *δ*_Sn_ = −374 ppm with a matching coupling constant (^1^*J*_Sn–H_ = 1580 Hz) and showing single-bond correlation to the ^1^H signal at 4.23 ppm. Both the ^1^H and ^119^Sn NMR chemical shifts are consistent with the ranges previously reported for bis(aryl)- and bis(silyl)tin(iv) dihydrides (*δ*_H_ = 3.15 to 6.23 ppm, *δ*_Sn_ = −331 to −355 ppm), while the ^1^*J*_Sn–H_ coupling constant falls between the values reported for bis(aryl)tin(iv) dihydrides (^1^*J*_Sn–H_ = 1820 to 1750 Hz) and the bis(boryl)tin(iv) dihydride {(HCDippN)_2_B}_2_SnH_2_ (Scheme 2b; ^1^*J*_Sn–H_ = 1300 Hz).^27,38–40^ To provide additional evidence for the formation of a Sn(iv) dihydride, colourless crystals of (1)H_2_ (Fig. 1) were obtained by slow evaporation of solvent from a C_6_D_6_ solution at room temperature.

**Scheme 3:**
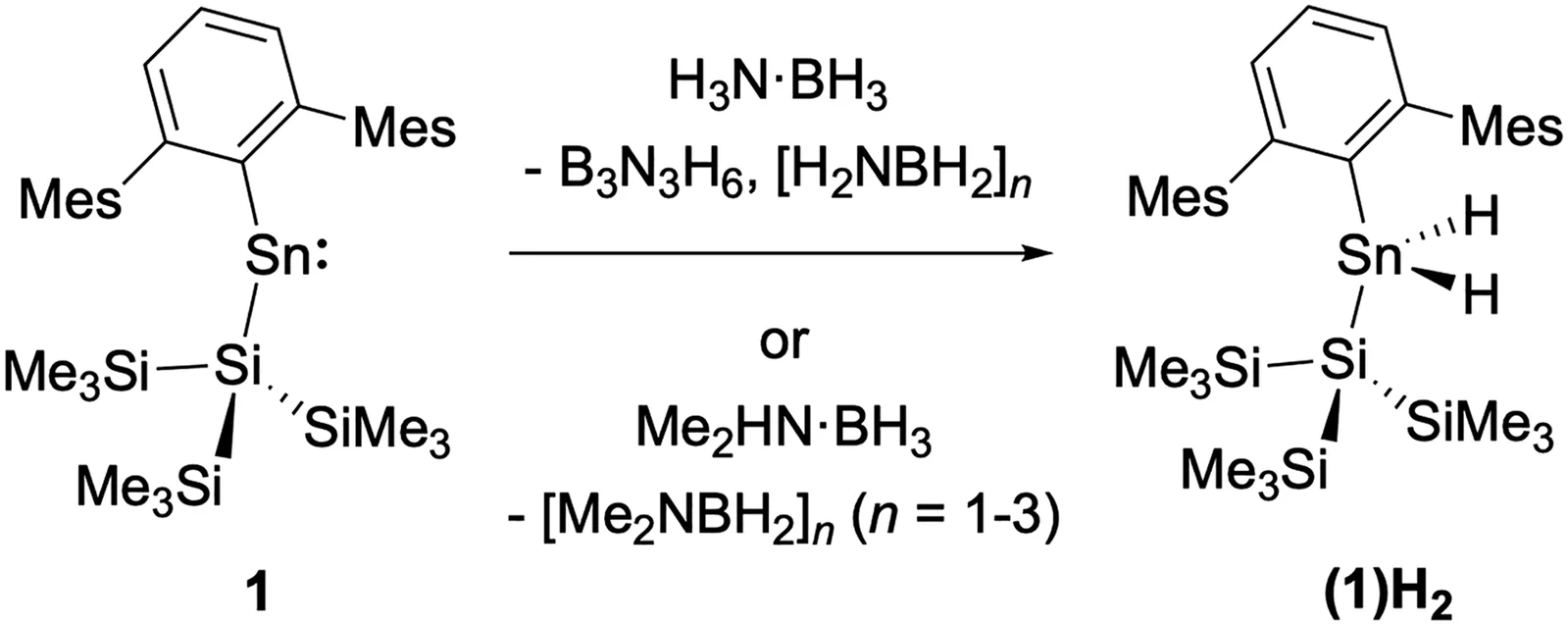
Reaction of stannylene 1 with ammonia borane (AB) or dimethylamineborane (DMAB) to form (1)H_2_.

**Fig. 1:**
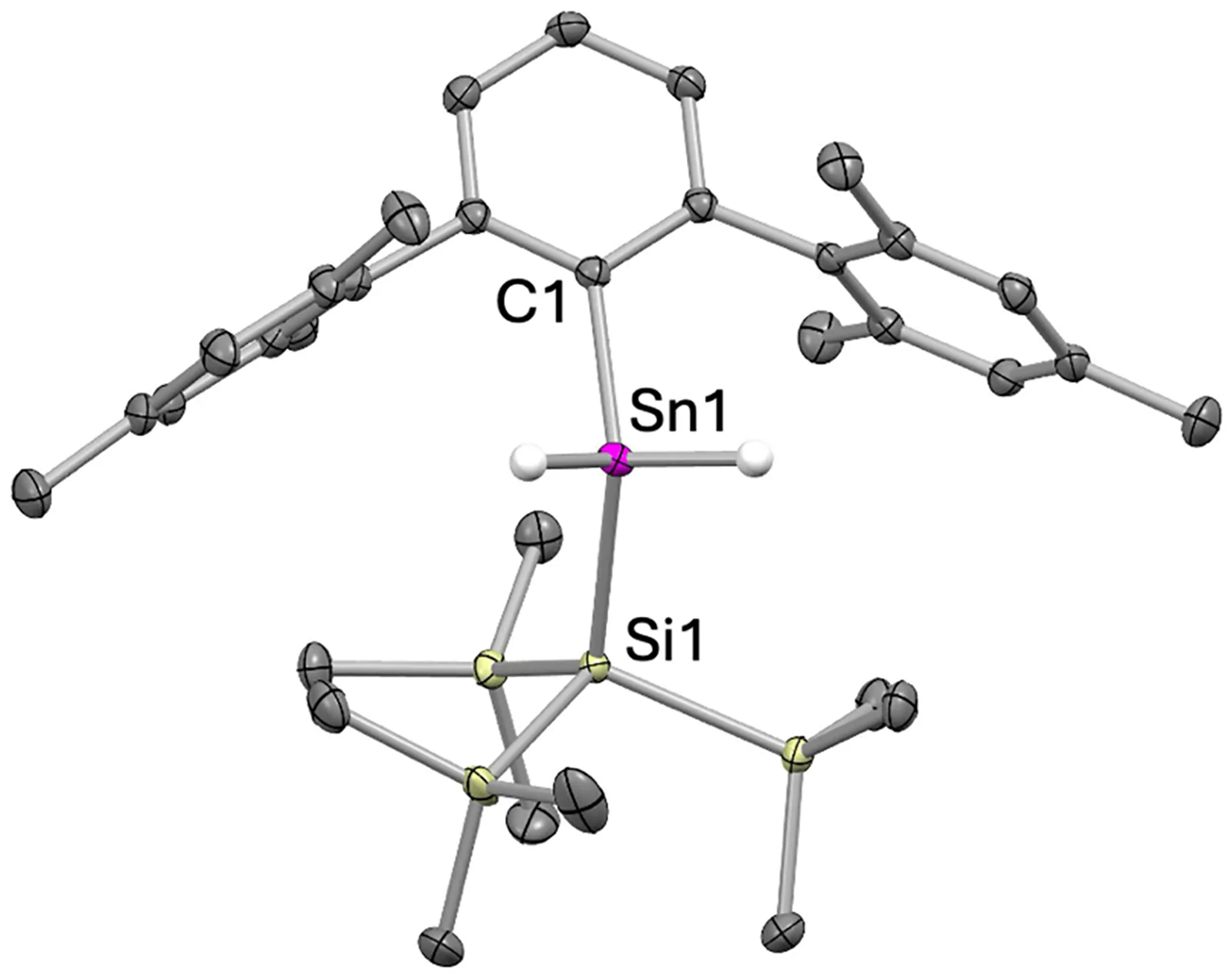
Molecular structure of (1)H_2_ as determined by X-ray crystallography. Thermal ellipsoids set at the 50% probability level; H atoms (except those bound to Sn) omitted for clarity. Key bond lengths (Å), angles (°): C1–Sn1 2.168(1), Sn1–Si1 2.5803(4), C1–Sn1–Si1 123.87(3).

Notwithstanding the well-known shortcomings of X-ray crystallography, the Sn-bound hydrogen atoms could be located in the difference Fourier map. More definitively, from a crystallographic perspective, the heavy atom skeleton is observed to widen on going from 1 to (1)H_2_, as expected due to the smaller size of the Sn(iv) centre formed on formal oxidation of Sn(ii).^27^ Accordingly, the C1–Sn1–Si1 bond angle (123.87(3)°) is significantly wider than that of 1 (109.75(6)°), and the C1–Sn1 and Si1–Sn1 distances are shorter (2.168(1) and 2.5803(4) Å, *cf*. 2.189(3) and 2.6407(7) Å, respectively).^30^ More broadly, the C1–Sn1 distance is within the range found for other known bis(aryl)tin(iv) dihydrides (2.154(1) to 2.172(2) Å), while the Sn1–Si1 distance (2.5803(4) Å) is comparable to those found for a cyclic bis(silyl)tin(iv) dihydride system reported by Zhao *et al.* (2.584(2) and 2.589(2) Å).^38–40^

Mechanistically, the more facile delivery of dihydrogen to 1 from AB (over H_2_ itself) is thought to reflect the intrinsic polarity (H^*δ*+^/H^*δ*−^) of the N–H and B–H bonds, and near-simultaneous delivery of these (activated) components to the HOMO and LUMO, respectively, of the stannylene.^41^ Previous studies have shown that *N*-substituted amine boranes (RH_2_N·BH_3_ or R_2_HN·BH_3_) dehydrogenate at lower temperatures than the parent H_3_N·BH_3_.^42,43^ Accordingly the reaction of dimethylamine borane (Me_2_NH·BH_3_; DMAB) with a toluene solution of 1 at room temperature (rather than 50 °C) results in immediate decolourisation, with the ^1^H NMR spectrum of the reaction mixture showing conversion to the same tin-containing product, (1)H_2_ (Scheme 3). The corresponding *in situ* measured ^11^B{^1^H} NMR spectrum allows the B/N-containing products to be identified as [Me_2_NBH_2_]_3_, [Me_2_NBH_2_]_2_ and Me_2_NBH_2_ (at *δ*_B_ = 2, 5 and 38 ppm, respectively).^44^ The synthesis of (1)H_2_ on a preparative scale is rapid at ambient temperature, with spectroscopically pure samples on a 100–200 mg scale obtainable directly from the reaction mixture by concentration of the toluene solvent and storage at −30 °C. With a facile route to (1)H_2_ in hand, we sought to probe its potential for H_2_ loss, either directly (*via* reductive elimination) or as a hydrogen transfer reagent to unsaturated substrates.

### Use of (1)H_2_ as a hydrogen transfer reagent

In our hands (1)H_2_ does not spontaneously evolve H_2_ under thermal activation: heating a solution of (1)H_2_ in C_6_D_6_ at 80 °C for 3 d shows no change, as determined by *in situ*^1^H NMR monitoring. In addition, no reaction is observed with phenylacetylene at 80 °C. Stannylene 1 has been shown previously to react rapidly with PhCCH at room temperature,^31^ suggesting that even if minute concentrations of 1 were formed from (1)H_2_*via* dehydrogenation, the alkyne would act as an effective stannylene trap. In the event, no (insertion) trapping product was detected.

Given the lack of reactivity both of 1 towards H_2_, and of (1)H_2_ towards the evolution of dihydrogen, we sought to probe both the thermodynamics and kinetics of hydrogen shuttling using quantum chemistry (Fig. 2). DFT studies (using the r^2^SCAN-3c method) imply that the ‘forward’ reaction (*i.e.* the oxidative addition of H_2_ at the Sn(ii) centre in 1) is mildly exergonic (but close to thermodynamically neutral) – consistent with the ability of the strongly σ-donating silyl ligand (in particular) to stabilise the Sn(iv) state. On the other hand, the activation barrier – as defined by a transition state in which an elongated H_2_ moiety is positioned across the formally vacant Sn 5p_*z*_ orbital – is prohibitively high (>35 kcal mol^−1^ in either direction). By means of comparison, bis(boryl)stannylene {(HCDippN)_2_B}_2_Sn has been shown to activate H_2_ at temperatures as low as −30 °C, implying a significantly lower activation barrier, which can be related in turn to the very low HOMO–LUMO energy separation associated with the presence of two strongly electron donating boryl substituents.^27,35^

**Fig. 2:**
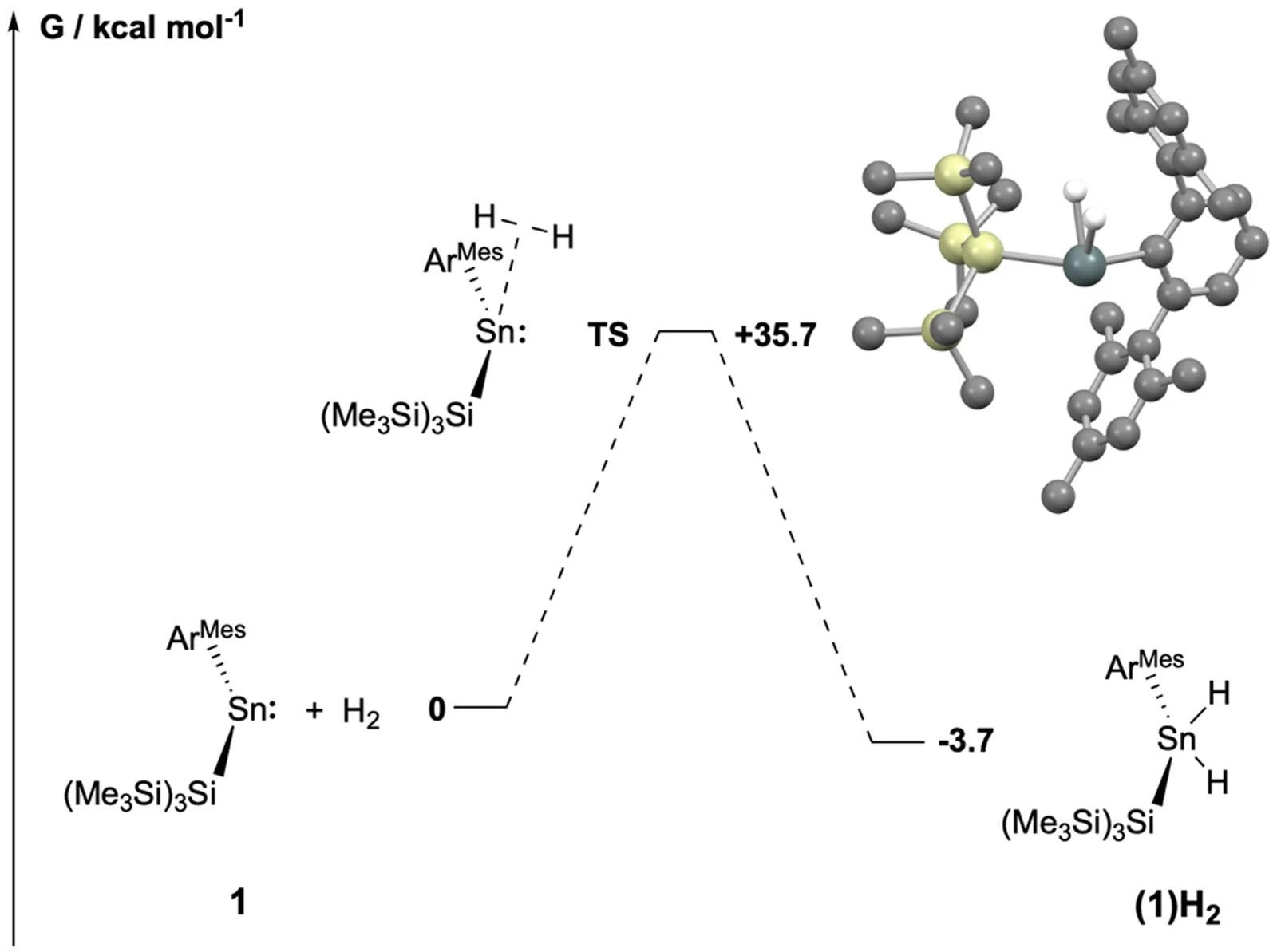
DFT calculated reaction pathway for the potential activation of dihydrogen by stannylene 1.

Sindlinger and Wesemann have shown that N-heterocyclic carbenes (NHCs) can be used to abstract dihydrogen from aryltin(iv) hydrides to generate low-valent tin species (and the corresponding hydrogenated carbene).^45^ With a view to probing whether (1)H_2_ could transfer H_2_ under similar conditions, we examined its reactivity towards carbene substrates. Combining (1)H_2_ and two equivalents of the sterically unencumbered NHC IMe_4_ (1,3,4,5-tetramethylimidazol-2-ylidene; Scheme 4) in C_6_D_6_ at room temperature results in the immediate formation of a pale-yellow solution. The ^1^H NMR spectrum measured *in situ* shows resonances for the hydrogenated carbene, *i.e.* 1,3,4,5-tetramethyl-2,3-dihydro-1*H*-imidazole, (IMe_4_)H_2_ (*δ*_H_ = 3.62, 2.22 and 1.53 ppm), together with a new set of Ar^Mes^/Si(SiMe_3_)_3_ signals differing from those of both 1 and (1)H_2_.^30,46,47^

**Scheme 4:**
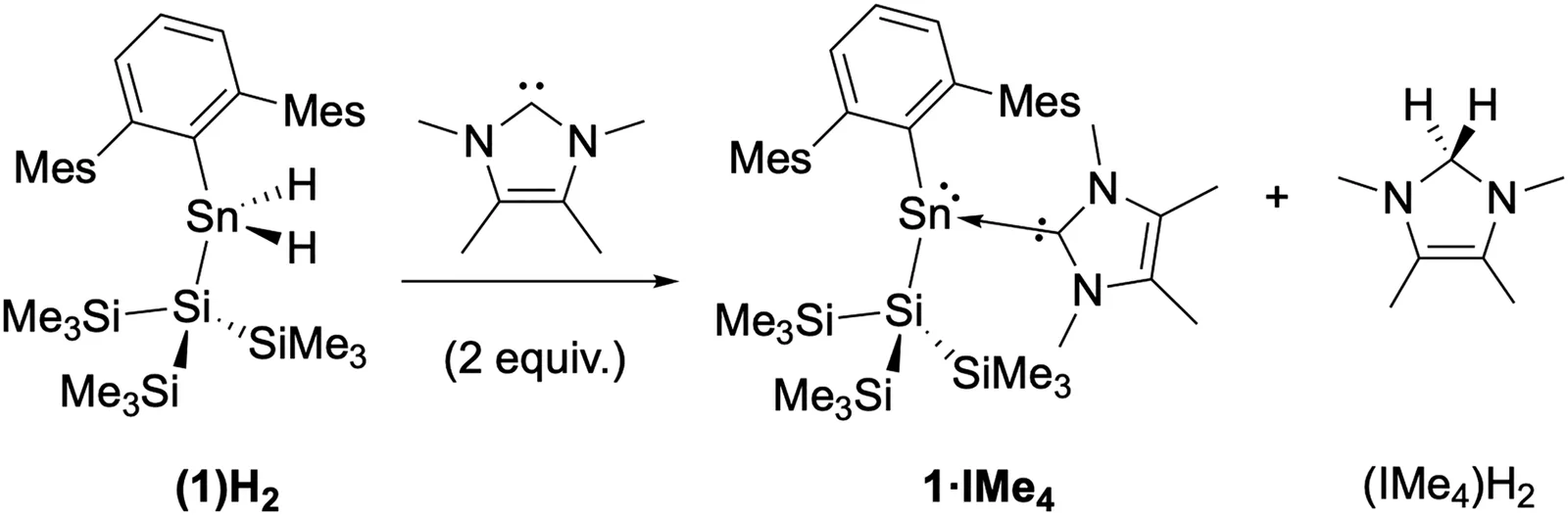
Reaction of (1)H_2_ with two equivalents of the NHC IMe_4_ (1,3,4,5-tetramethylimidazol-2-ylidene) to form adduct 1·IMe_4_ and (IMe_4_)H_2_ (1,3,4,5-tetramethyl-2,3-dihydro-1*H*-imidazole).

Hypothesising that the other product of this reaction is a stannylene-carbene adduct, the independent reaction of 1 with IMe_4_ was investigated (Scheme 5). Combination of these two reagents in C_6_D_6_ solution results in an immediate colour change to pale yellow. The resonances observed by ^1^H NMR spectroscopy match those of the product formed in the reaction of (1)H_2_ with IMe_4_, which is therefore proposed to be 1·IMe_4_, *i.e.* the donor/acceptor adduct of 1 and IMe_4_ (Schemes 4 and 5). Consistent with this hypothesis, the reaction of (1)H_2_ with IMe_4_ requires two equivalents of carbene to be driven to completion; reactions involving fewer equivalents yield mixtures of (IMe_4_)H_2_, 1·IMe_4_ and unreacted (1)H_2_. Tin-to-carbon transfer of H_2_ is therefore shown to be accompanied by sequestration of ‘free’ 1 by IMe_4_.

**Scheme 5:**
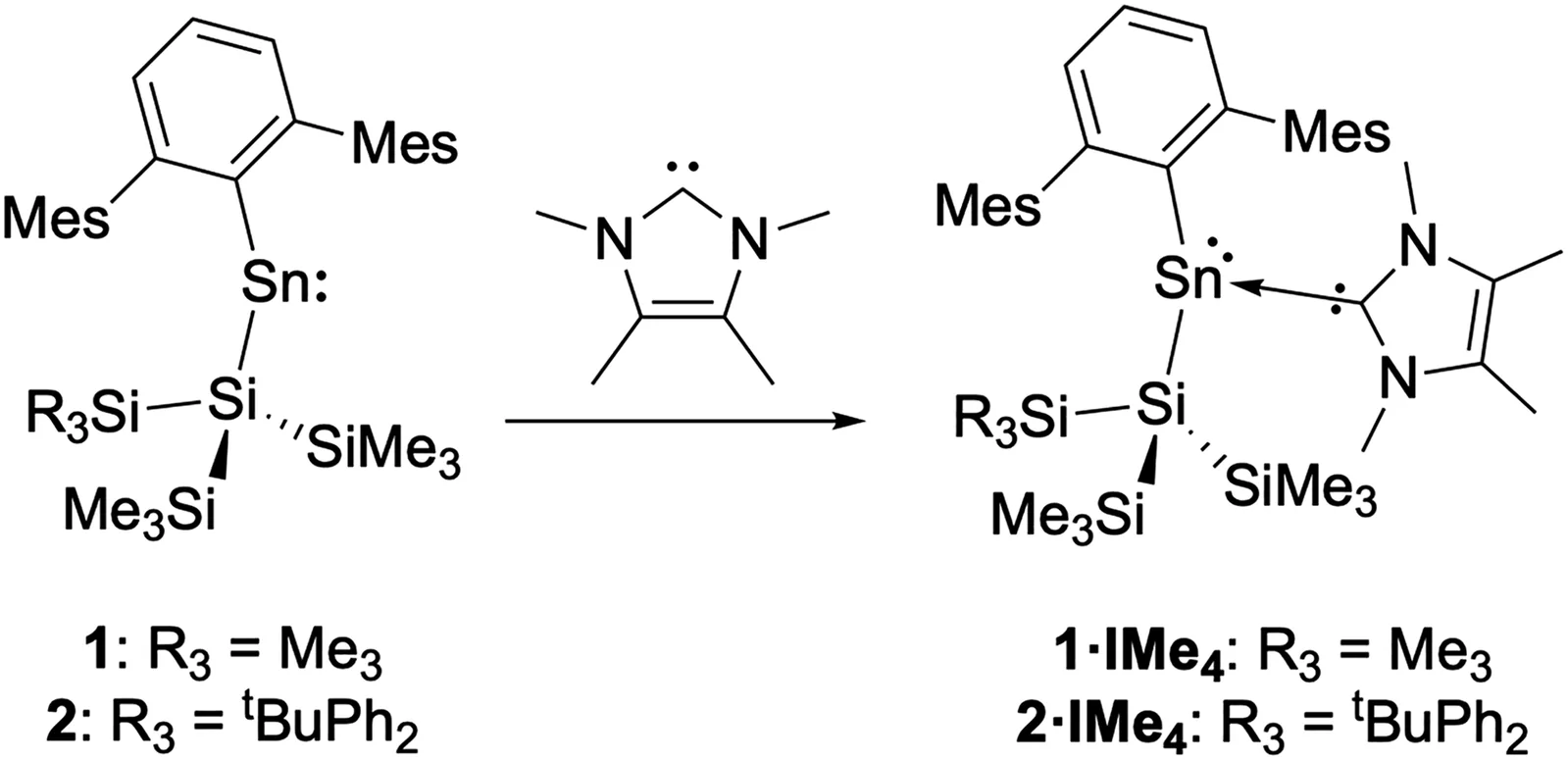
Reaction of stannylenes 1 and 2 with IMe_4_ to form adducts 1·IMe_4_ and 2·IMe_4_, respectively.

1·IMe_4_ has been characterized by multinuclear NMR spectroscopy. Restricted rotation of both the carbene and Mes rings in 1·IMe_4_ is suggested in the ^1^H NMR spectrum by the presence of two distinct NCH_3_ signals (at *δ*_H_ = 3.37 and 2.80 ppm) and three Mes CH_3_ singlets (at *δ*_H_ = 2.45, 2.25 and 1.99 ppm), and presumably reflects enhanced steric hindrance on assimilation of the carbene donor. The corresponding ^119^Sn NMR spectrum shows a signal at *δ*_Sn_ = −140 ppm, which lies close to the range defined by IMe_4_ adducts of aryl(alkyl)- and bis(silyl)-substituted stannylenes (*δ*_Sn_ = −164 to −466 ppm).^48–55^ While 1·IMe_4_ could not be crystallised, the closely related stannylene Ar^Mes^Sn{Si(SiMe_3_)_2_(Si^*t*^BuPh_2_)} (2; Scheme 5) was found to react with IMe_4_ in similar fashion, yielding a product with a similar ^119^Sn NMR shift to that of 1·IMe_4_ (at *δ*_Sn_ = −95 ppm).^32,56^ In this case, colourless crystals of the adduct 2·IMe_4_ (Fig. 3) suitable for X-ray crystallography could be obtained by slow evaporation of solvent from a pentane solution at room temperature. X-ray crystallography confirms the formation of the adduct 2·IMe_4_, featuring a pyramidal geometry around Sn (sum of angles: 308.1(1)°). Carbene coordination using the vacant Sn 5p_*z*_ orbital of 2 is implied by the nearly perpendicular C1–Sn1–C2 and Si1–Sn1–C2 angles (99.63(9) and 93.83(7)°, respectively). The C2–Sn1 distance of 2.306(3) Å is within the range for donor/acceptor IMe_4_ adducts of aryl(alkyl)- or bis(silyl)-substituted stannylenes (range 2.25(1)–2.316(2) Å). The aryl C1–Sn1 distance (2.273(3) Å) is also within the precedented range of Sn–C_alkyl_ and Sn–C_aryl_ distances (range 2.242(1)–2.311(1) Å), while the Sn1–Si1 distance (2.773(1) Å) is slightly longer (range 2.6247(7)–2.713(4) Å).^48–55^ Compared to the free stannylene 2, the C1–Sn1 and Sn1–Si1 distances (2.273(3) and 2.773(1) Å, respectively) are slightly extended (2.220(3) and 2.6807(9) Å), and the C1–Sn1–Si1 angle is marginally wider (114.68(7) *cf*. 113.96(8)°), presumably due to enhanced steric factors.^32^ Structurally, the geometry of 2·IMe_4_ in the solid state also appears to be influenced by π–π stacking interactions between one of the flanking Mes groups and the NHC heterocycle, as reflected in a Mes_centroid_⋯Ar_centroid_ distance of 3.309 Å.^48–55^

**Fig. 3:**
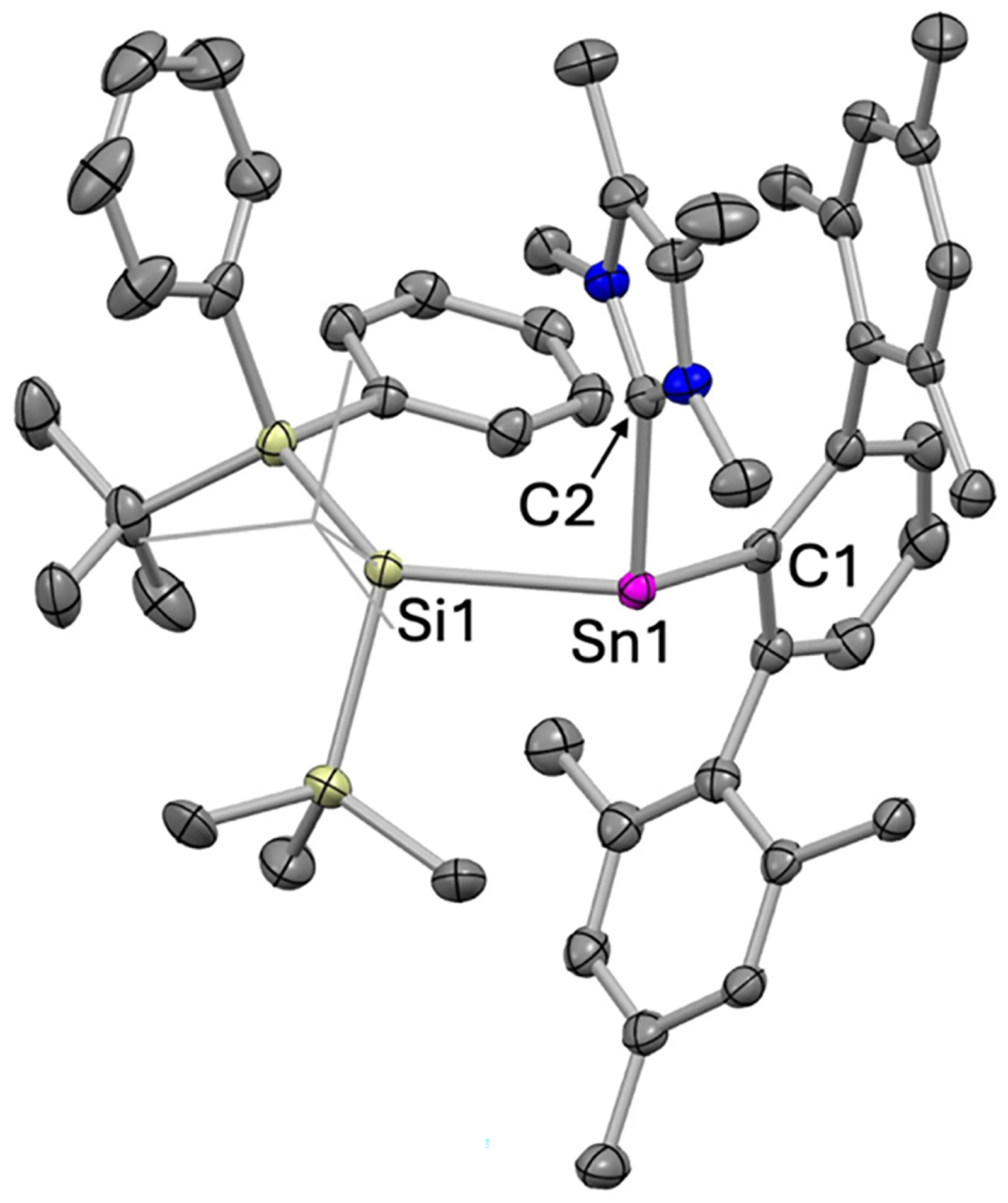
Structure of 2·IMe_4_ as determined by X-ray crystallography. Thermal ellipsoids set at the 50% probability level; H atoms omitted and one SiMe_3_ group shown in wireframe for clarity. Key bond lengths (Å) and angles (°): C1–Sn1 2.273(3), C2–Sn1 2.306(3), Sn1–Si1 2.773(1), C1–Sn1–C2 99.63(9), C1–Sn1–Si1 114.68(7), C2–Sn1–Si1 93.83(7).

## Conclusions

In conclusion, stannylene 1 featuring a strongly electron donating silyl substituent has been shown to offer a close to thermodynamically neutral platform for the shuttling of H_2_ (Δ*G*° = −3.7 kcal mol^−1^ for H_2_ uptake). While the direct activation and release of dihydrogen itself is precluded by prohibitively high kinetic barriers (Δ*G*^‡^ = −35.7 kcal mol^−1^), 1 can take up the elements of H_2_ to form tin(iv) dihydride (1)H_2_ by the dehydrogenation of both ammonia- and dimethylamine-borane. (1)H_2_ in turn, is shown to be competent for transfer hydrogenation to an NHC, to yield the corresponding dihydro-imidazole and regenerate the Sn(ii) state in the form of the stannylene-carbene adduct 1·IMe_4_.

## Conflicts of interest

The authors declare no competing interests.

## Supplementary Material

DT-055-D6DT02289H-s001

## Data Availability

The original data on which this publication is based are included in the supplementary information (SI). Supplementary information: experimental procedures, characterizing data, representative spectra, crystallographic details and computational data. See DOI: https://doi.org/10.1039/d6dt02289h. CCDC 2588787 and 2588788 contain the supplementary crystallographic data for this paper.^57*a*,*b*^
