# Supplementary material for: Exploiting Sn(ii)/Sn(iv) redox chemistry: a silyl-stannylene offering close-to-thermoneutral transfer hydrogenation
Source: Dalton Trans. 2026 Oct 5;55(41):14913–8. doi: 10.1039/d6dt02289h (PMC13662779; doi:10.1039/d6dt02289h)
Supplement: Supplementary file 1 [file DT-055-D6DT02289H-s001.pdf]

Supplementary Information (SI) for Dalton Transactions.
This journal is © The Royal Society of Chemistry

Exploiting Sn(II)/Sn(IV) Redox Chemistry: a Silyl-Stannylene Offering Close-to-Thermoneutral Transfer Hydrogenation

Aidan J. Murray, Agamemnon E. Crumpton and Simon Aldridge*

Supporting Information (20 pages):

1. General Considerations S2
2. Syntheses of Novel Compounds S3
3. X-ray Crystallographic Details S5
4. NMR Spectra of Reaction Mixtures S6
5. NMR Spectra of Novel Compounds S7
6. Quantum Chemical Calculations S15
7. References S20

1. General Considerations

All manipulations were performed using standard Schlenk line or dry-box techniques under an argon or nitrogen atmosphere. Solvents were degassed by sparging with argon and dried by passing through a column of the appropriate drying agent, then stored under argon in Teflon-valve ampoules over a potassium mirror or 3 Å molecular sieves. NMR spectra were measured in benzene-d_6 (C_6D_6) which was pre-dried over CaH_2, distilled under reduced pressure and stored under argon in Teflon valve ampoules over 3 Å molecular sieves. NMR samples were prepared under argon in 5 mm Wilmad 507-PP tubes fitted with J. Young Teflon valves. NMR spectra were measured on a Bruker Avance III HD Nanobay 400 MHz NMR spectrometer or a Bruker Avance III 500 MHz NMR spectrometer. Unless otherwise stated, all ^{11}B, ^{13}C, ^{29}Si and ^{119}Sn NMR measurements were performed with proton decoupling. ^{1}H and ^{13}C NMR spectra were referenced internally to residual protio-solvent (^{1}H) or solvent (^{13}C) resonances which are reported relative to $SiMe_4$. ^{11}B, ^{29}Si and ^{119}Sn NMR spectra were referenced externally with respect to $BF_3 \cdot OEt_2$, $SiMe_4$ and $SnMe_4$, respectively. Chemical shifts are quoted in δ (ppm) and coupling constants in Hz. 2D NMR techniques (COSY, HSQC, and HMBC) were used to aid in assignment of spectra. Hydrogen gas was dried over P_2O_5, with all other reagents used as received. The known compounds **1**, **2** and IMe_4 were prepared according to existing literature procedures, with matching spectroscopic data.[s1–s3]

2. Syntheses of Novel Compounds

(1)H$_2$: **1** (250 mg, 0.37 mmol) and $Me_2HN{\cdot}BH_3$ (26 mg, 0.44 mmol) were combined as solids and the mixture dissolved in toluene (10 mL). The reaction mixture was stirred at room temperature for 1 h, over which time the solution turned from green to colourless. Concentration of the solution to *ca*. one third of its original volume and storage at −30 °C overnight afforded the product **(1)H$_2$** as a white powder, which was isolated by removal of the mother liquor *via* a thin cannula. Colourless crystals of quality suitable for X-ray crystallography were obtained by slow evaporation of solvent from a C_6D_6 solution at room temperature. Yield: 146 mg, 58%.

Calc. for $C_{33}H_{54}Si_4Sn$: C 58.13%, H 7.98%. Meas.: C 58.19%, H 7.45%.

^{1}H NMR (400 MHz, C_6D_6, 298 K): δ_H 7.23 (t, $^3J_{HH}$ = 7.5 Hz, 1H, *p*-CH Ar^{Mes}), 6.97 (d, $^3J_{HH}$ = 7.5 Hz, 2H, *m*-CH Ar^{Mes}), 6.88 (s, 4H, *m*-CH Mes), 4.23 (s, $^{119/117}$Sn satellites: $^1J_{Sn-H}$ = 1580, 1510 Hz, 2H, SnH_2), 2.27 (s, 6H, *p*-CH_3 Mes), 2.15 (s, 12H, *o*-CH_3 Mes), 0.17 (s, 27H, $Si(Si(CH_3)_3)_3$) ppm.

^{13}C{^{1}H} NMR (101 MHz, C_6D_6, 298 K): δ_C 151.2, 142.6, 140.4, 136.9, 135.8, 129.7, 128.9, 127.7, 21.8, 21.2, 2.7 ppm.

^{29}Si{^{1}H} NMR (80 MHz, C_6D_6, 298 K): δ_{Si} 72.6, −8.0 ppm (assigned by ^{29}Si/^{1}H HMBC).

^{119}Sn NMR (186 MHz, C_6D_6, 298 K): δ_{Sn} −374 (t, $^1J_{Sn-H}$ = 1580 Hz) ppm.

1·IMe$_4$: In a J Young's NMR tube, **1** (27 mg, 0.040 mmol) and IMe_4 (5 mg, 0.040 mmol) were combined as solids and the mixture dissolved in C_6D_6 (0.5 mL), with immediate formation of a pale-yellow solution. From this mixture, the product **1·IMe$_4$** was characterised spectroscopically *in situ*.

^{1}H NMR (400 MHz, C_6D_6, 298 K): δ_H 7.12 (t, $^3J_{HH}$ = 7.5 Hz, 1H, *p*-CH Ar^{Mes}), 6.84 (s, 2H, *m*-CH Mes), 6.78 (d, $^3J_{HH}$ = 7.5 Hz, 2H, *m*-CH Ar^{Mes}), 6.68 (s, 2H, *m*-CH Mes), 3.37 (s, 3H, NCH_3), 2.80 (s, 3H, NCH_3), 2.45 (s, 6H, CH_3 Mes), 2.25 (s, 6H, CH_3 Mes), 1.99 (s, 6H, CH_3 Mes), 1.39 (s, 6H, $CH_3C{=}CCH_3$), 0.24 (s, 27H, $Si(Si(CH_3)_3)_3$) ppm.

^{13}C{^{1}H} NMR (101 MHz, C_6D_6, 298 K): δ_C 174.7, 160.4, 150.3, 144.6, 136.3, 136.2, 135.3, 129.5, 129.2, 128.8, 126.3, 26.9, 22.8, 21.8, 21.3, 8.5, 4.9 ppm.

^{29}Si{^{1}H} NMR (80 MHz, C_6D_6, 298 K): δ_{Si} −7.3, −128.2 ppm.

^{119}Sn{^{1}H} NMR (150 MHz, C_6D_6, 298 K): δ_{Sn} −140 ppm.

2·IMe$_4$: **2** (107 mg, 0.13 mmol) and IMe_4 (16 mg, 0.13 mmol) were combined as solids and the mixture dissolved in toluene (3 mL), with immediate formation of a dark red solution. After stirring at room

temperature for 30 min, volatiles were removed *in vacuo* to leave an oily red residue. This residue was dissolved in pentane (2 mL), with rapid onset of crystallisation. Storage at room temperature for 1 h yielded product **2·IMe$_4$** as an orange powder, which was isolated by removal of the mother liquor *via* a thin cannula. Colourless crystals of quality suitable for X-ray crystallography were obtained by slow evaporation of solvent from a pentane solution at room temperature. Yield: 51 mg, 40%.

Calc. for $C_{53}H_{74}N_2Si_4Sn$: C 65.61%, H 7.69%, N 2.89%. Meas.: C 64.70%, H 7.09%, N 2.29%.

^{1}H NMR (400 MHz, C_6D_6, 298 K): δ_H 8.03 – 7.99 (m, 2H, CH Ph), 7.88 – 7.84 (m, 2H, CH Ph), 7.22 – 7.08 (m, 7H, ArH), 6.84 (d, $^{3}J_{HH}$ = 7.5 Hz, 2H, *m*-CH Ar^{Mes}), 6.75 (s, 2H, *m*-CH Mes), 6.63 (s, 2H, *m*-CH Mes), 2.97 (br. s, 6H, NCH_3), 2.25 (s, 6H, CH_3 Mes), 2.18 (s, 6H, CH_3 Mes), 2.16 (s, 6H, CH_3 Mes), 1.32 (s, 6H, $CH_3C{=}CCH_3$), 1.28 (s, 9H, $C(CH_3)_3$), 0.21 (s, 9H, $Si(CH_3)_3$), 0.02 (s, 9H, $Si(CH_3)_3$) ppm.

$^{13}C\{^{1}H\}$ NMR (101 MHz, C_6D_6, 298 K): δ_C 174.6, 163.3, 151.0, 144.2, 141.0, 139.5, 138.4, 137.8, 136.5, 136.5, 135.4, 129.6, 129.0, 128.7, 128.5, 127.5, 127.2, 126.6, 125.4, 31.0, 22.9, 22.5, 21.5, 21.2, 8.4, 6.2, 5.6 ppm.

$^{29}Si\{^{1}H\}$ NMR (80 MHz, C_6D_6, 298 K): δ_{Si} 4.9, −6.8, −123.9 ppm.

$^{119}Sn\{^{1}H\}$ NMR (150 MHz, C_6D_6, 298 K): δ_{Sn} −95 ppm.

3. X-ray Crystallographic Details

Single-crystal X-ray diffraction data were collected on a Rigaku XtaLAB Synergy-DW VHF equipped with a PhotonJet-R dual wavelength rotating anode and HyPix-Arc 150° detector. Crystals were selected under Paratone-N oil, mounted on MiTeGen Micromount loops and quench-cooled using an Oxford Cryosystems open flow N_2 cooling device.[s4] Data were collected at 100 K using mirror monochromated Mo Kα (λ = 0.71073 Å) or Cu Kα radiation (λ = 1.5418 Å). Data collected were processed using the CrysAlisPro package. Structures were solved *ab initio* from the integrated intensities using SHELXT and refined on F^2 using SHELXL with the graphical interface OLEX2.[s5–s7]

	**(1)H₂**	**2·IMe₄**
**Empirical Formula**	$C_{33}H_{54}Si_4Sn$	$C_{53}H_{74}N_2Si_4Sn$
**Fw (g mol⁻¹)**	681.81	970.19
**Temperature (K)**	99.99(10)	99.98(18)
**Crystal system**	monoclinic	orthorhombic
**Space group**	$P2_1/n$	Pbca
**a (Å)**	11.60780(10)	14.61390(10)
**b (Å)**	19.28980(10)	17.45710(10)
**c (Å)**	16.36680(10)	40.3187(3)
**α (°)**	90	90
**ß (°)**	91.6280(10)	90
**λ (°)**	90	90
**V (Å⁻³)**	3663.25(4)	10285.96(12)
**Z**	4	8
**ρ_{calc} (g cm⁻³)**	1.236	1.253
**μ (mm⁻¹)**	0.848	5.105
**F(000)**	1432.0	4096.0
**Crystal size (mm⁻³)**	0.3 × 0.2 × 0.2	0.2 × 0.05 × 0.05
**Radiation, λ (Å)**	0.71073	1.54184
**2Θ range for data collection (°)**	4.094 to 54.968	4.384 to 150.296
**Index ranges**	−15 ≤ h ≤ 15, −25 ≤ k ≤ 25, −21 ≤ l ≤ 21	−18 ≤ h ≤ 18, −21 ≤ k ≤ 15, −48 ≤ l ≤ 47
**Reflections collected**	237737	99003
**Independent reflections**	8395 [R_{int} = 0.0206, R_{sigma} = 0.0090]	10175 [R_{int} = 0.0439, R_{sigma} = 0.0221]
**Data / restraints / parameters**	8395 / 0 / 366	10175 / 0 / 560
**Goodness-of-fit on F²**	1.061	1.044
**Final R indexes [I ≥ 2σ (I)]**	R_1 = 0.0185 ωR_2 = 0.0480	R_1 = 0.0281 ωR_2 = 0.0687
**Final R indexes [all data]**	R_1 = 0.0186 ωR_2 = 0.0481	R_1 = 0.0357 ωR_2 = 0.0716
**Largest diff. peak/hole (e Å⁻³)**	1.45 / −0.47	0.51 / −0.65
**CCDC ref**	2588788	2588787

Table S1. X-ray crystallographic details for compounds **(1)H₂** and **2·IMe₄**.

4. NMR Spectra of Reaction Mixtures

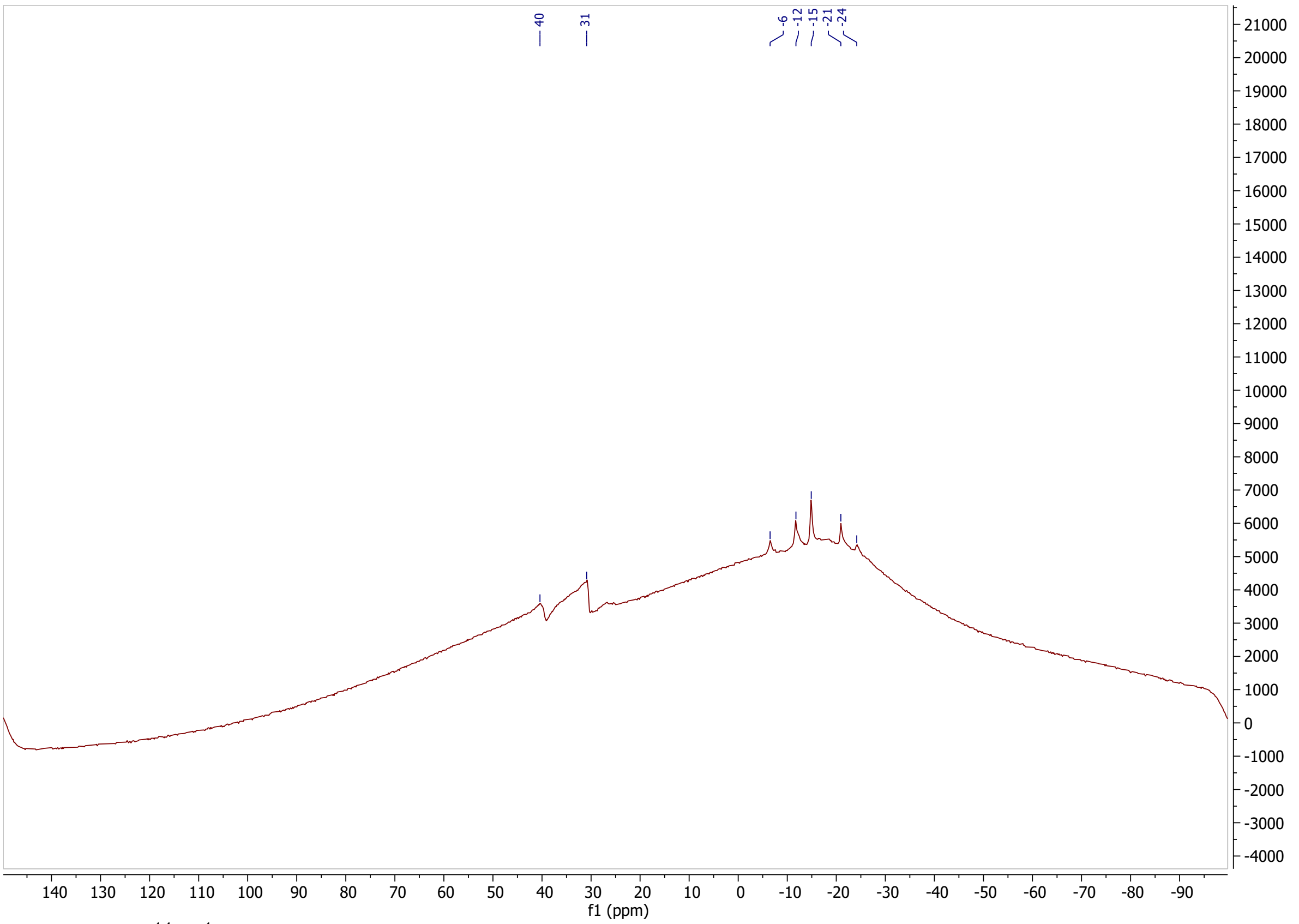

Figure S1. $^{11}B\{^1H\}$ NMR spectrum of the reaction mixture between **1** and excess AB in C_6D_6 at 298 K.

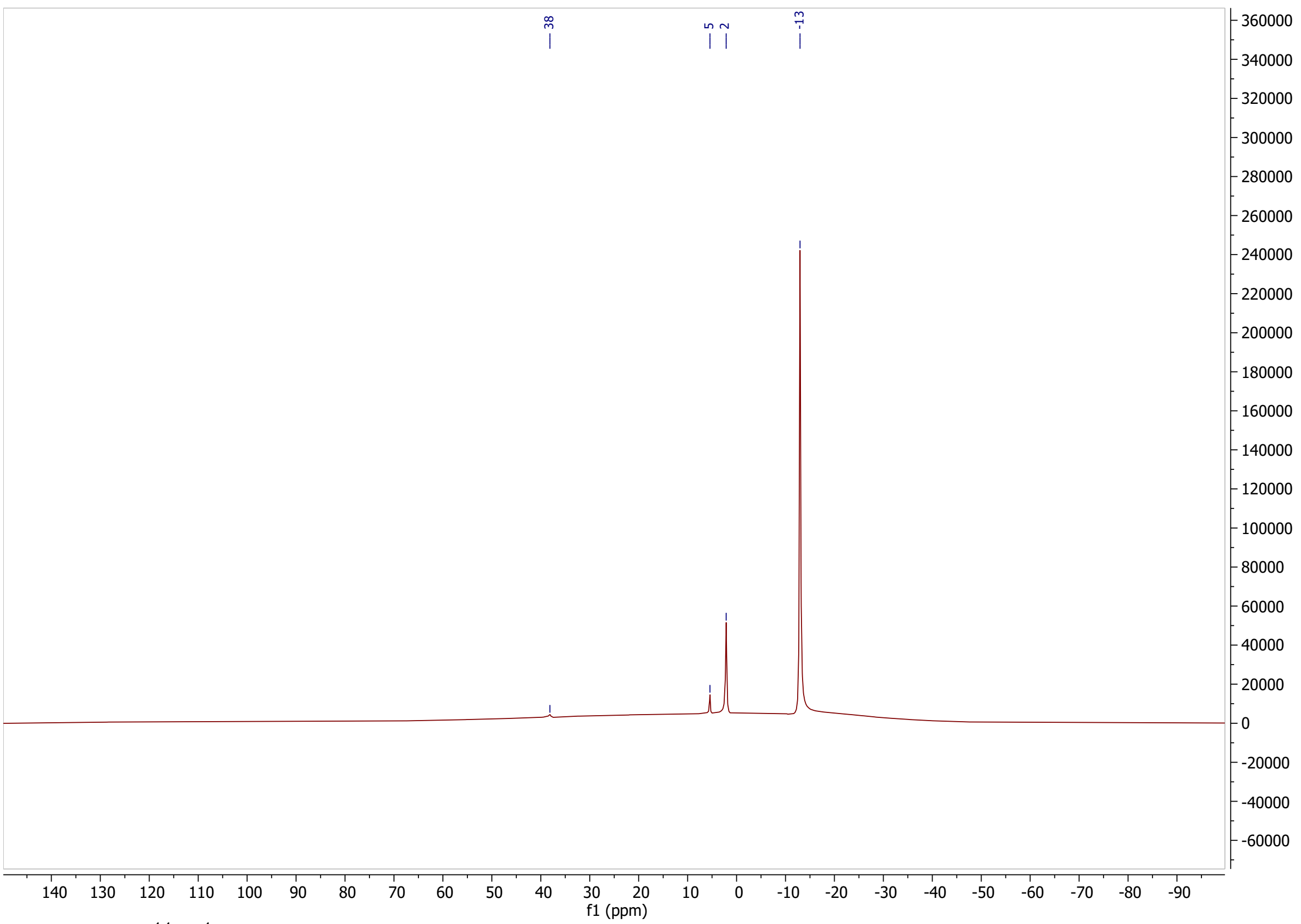

Figure S2. $^{11}B\{^1H\}$ NMR spectrum of the reaction mixture between **1** and excess DMAB in C_6D_6 at 298 K.

5. NMR Spectra of Novel Compounds

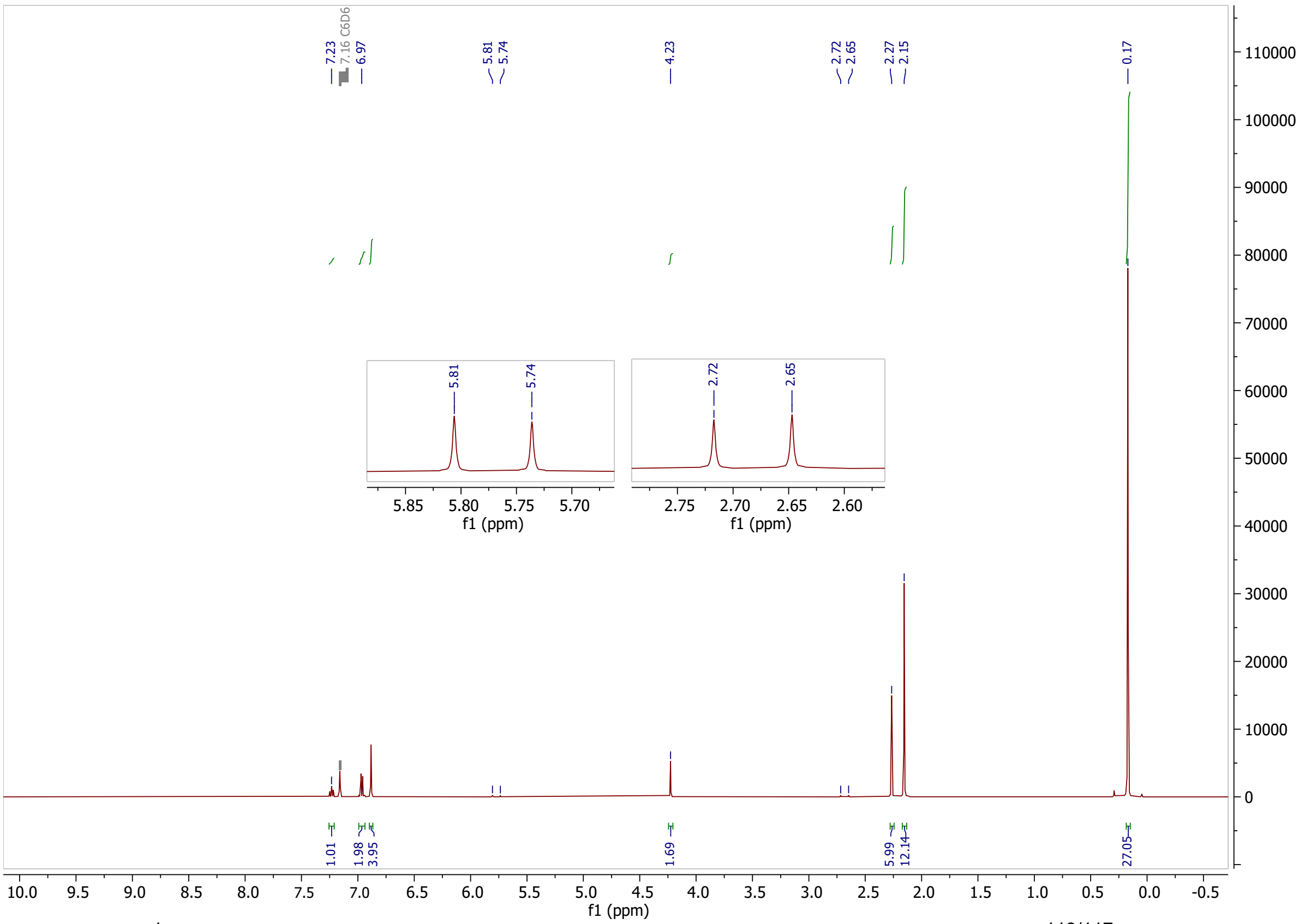

Figure S3. ^{1}H NMR spectrum of **(1)H$_2$** in C_6D_6 at 298 K, with inserts showing the $^{119/117}$Sn satellites.

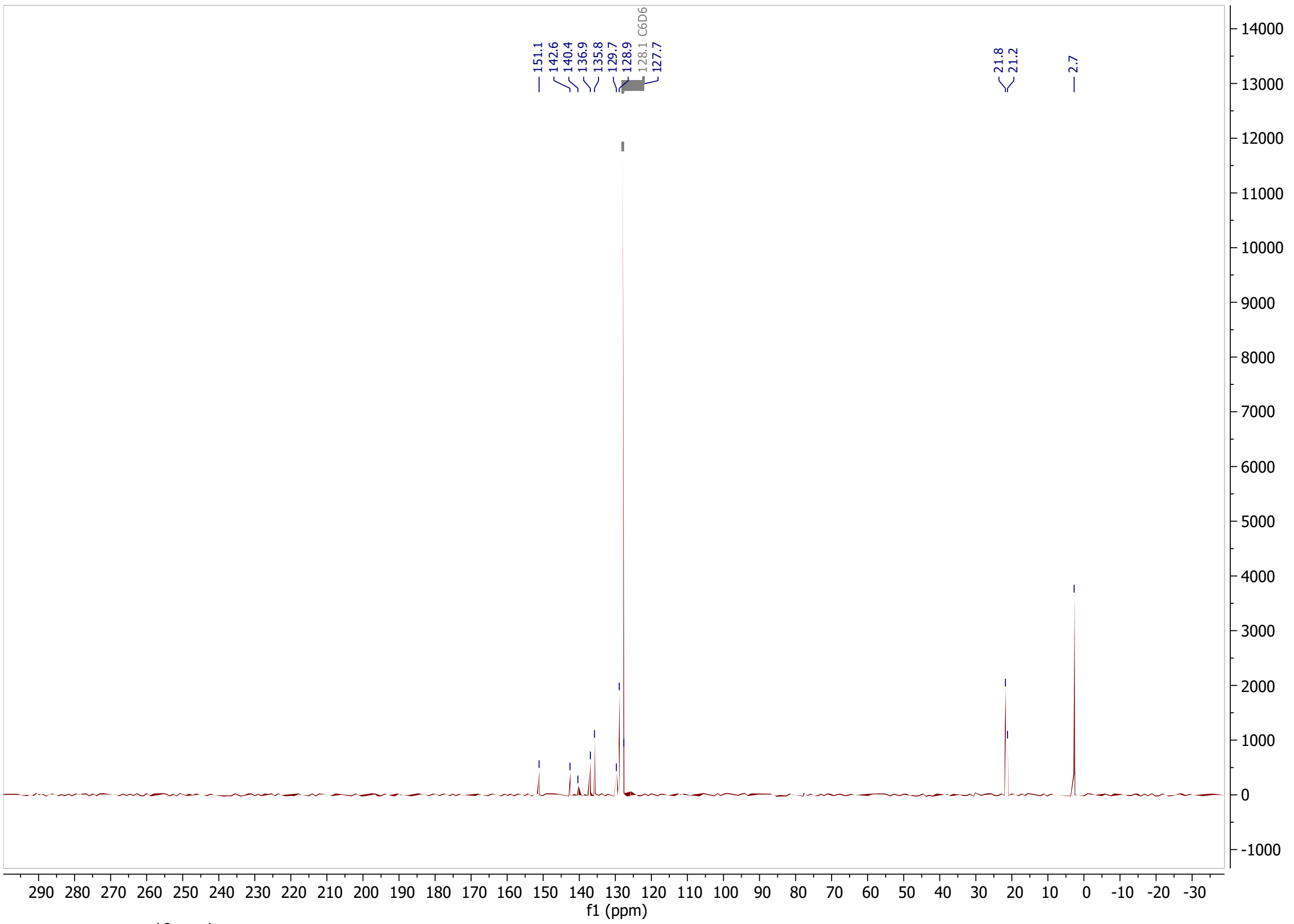

Figure S4. ^{13}C{^{1}H} NMR spectrum of **(1)H$_2$** in C_6D_6 at 298 K.

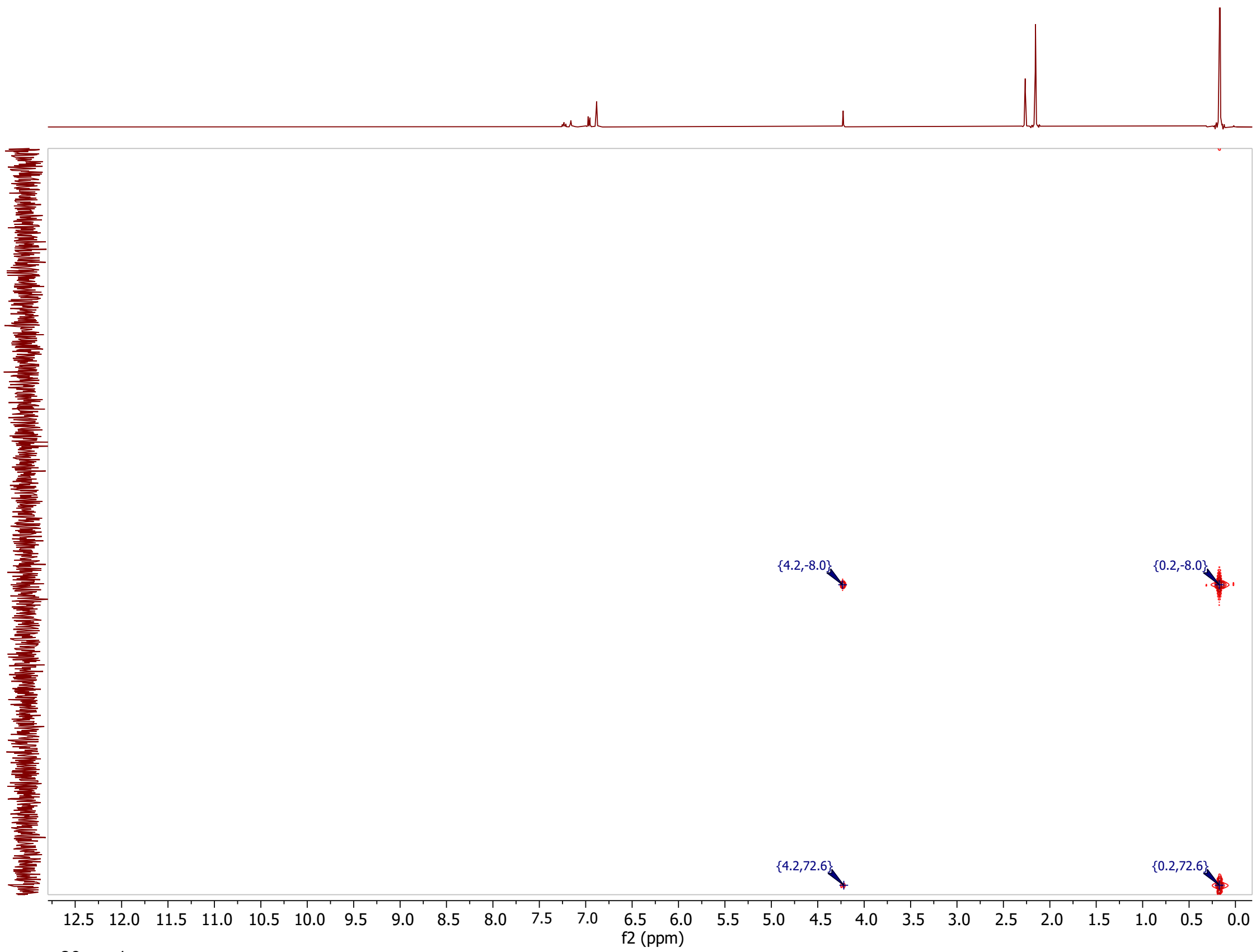

Figure S5. $^{29}Si/^{1}H$ HMBC NMR spectrum of **(1)H₂** in C_6D_6 at 298 K.

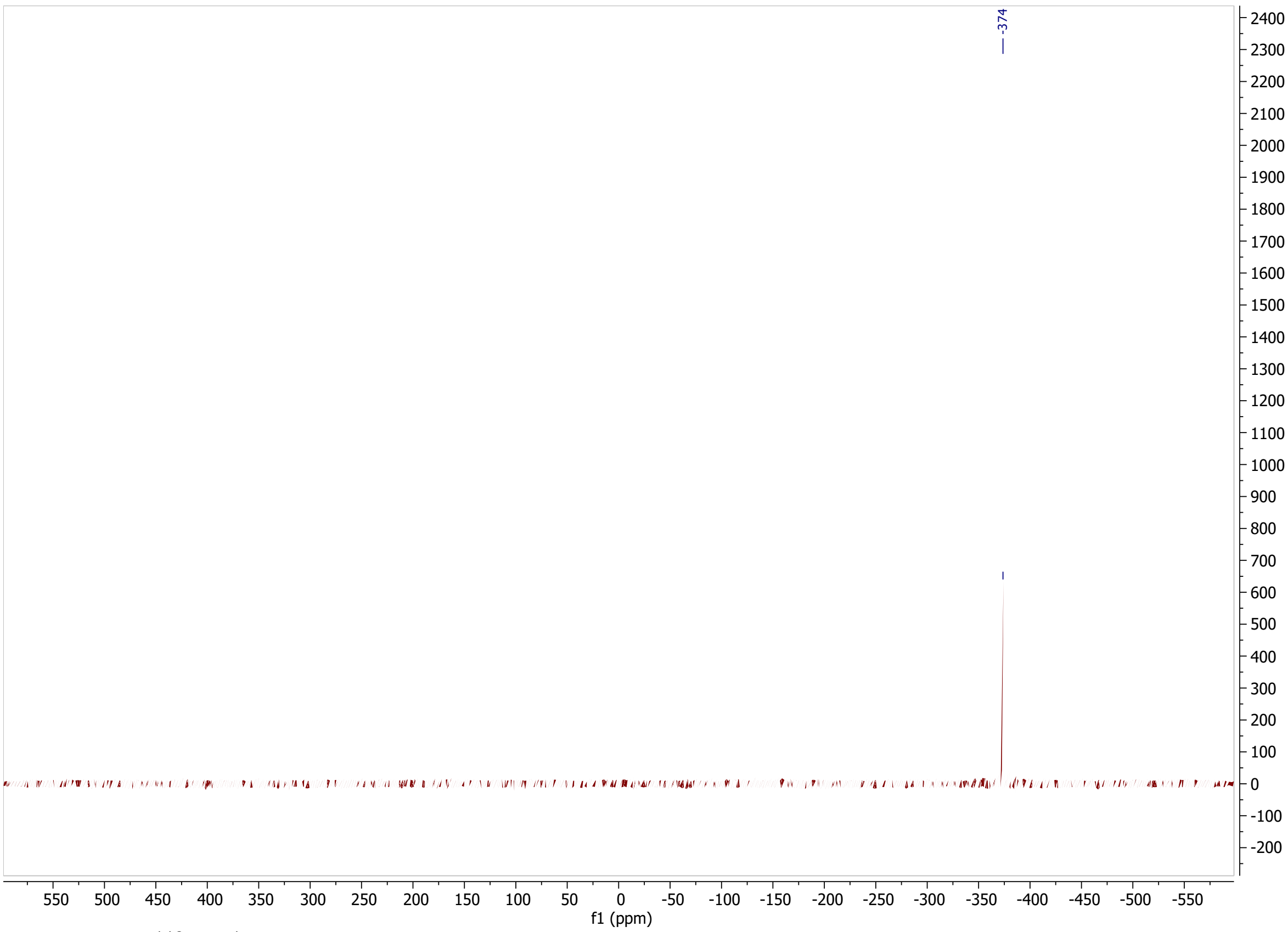

Figure S6. $^{119}Sn\{^{1}H\}$ NMR spectrum of **(1)H₂** in C_6D_6 at 298 K.

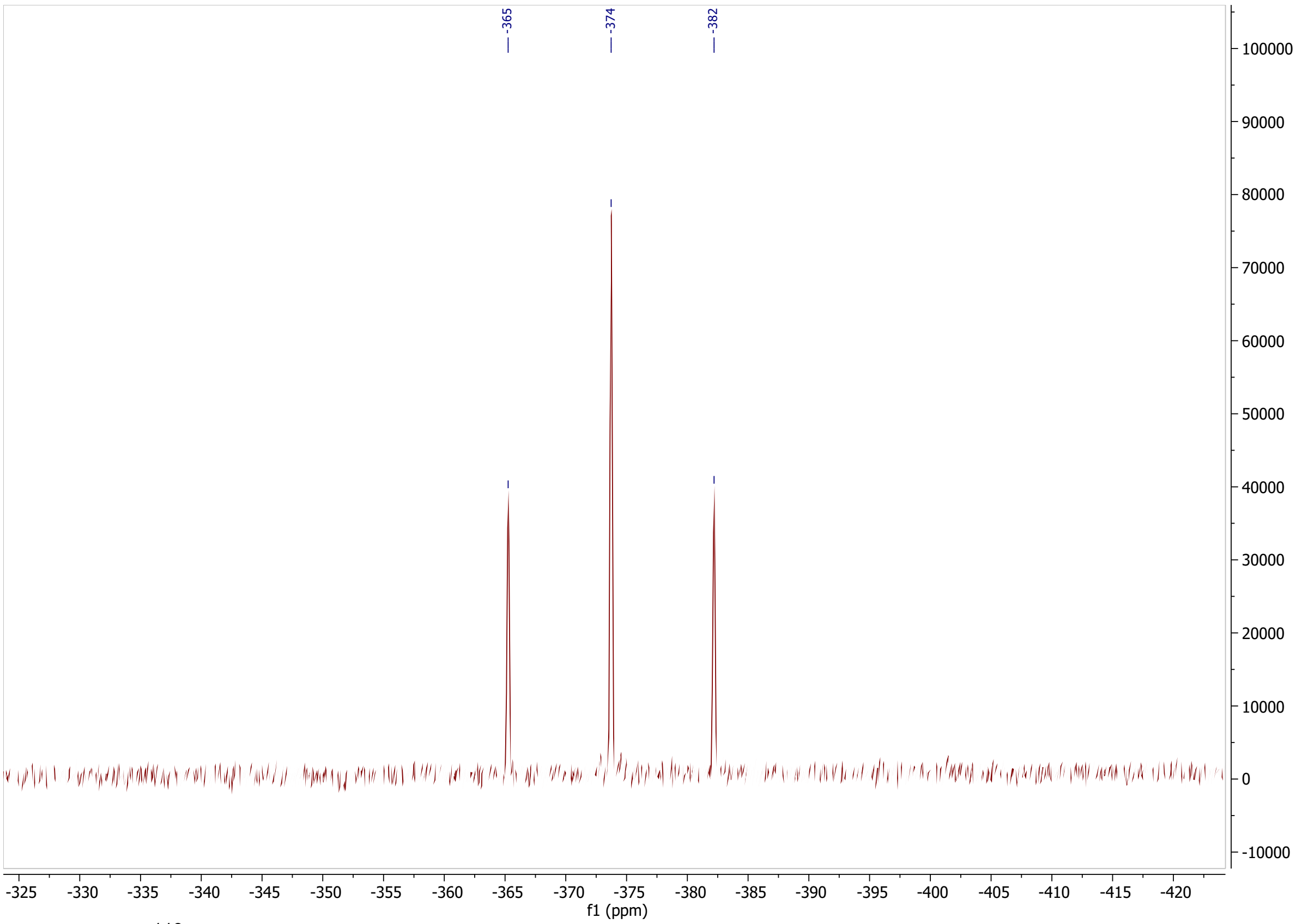

Figure S7. ^{119}Sn NMR spectrum of **(1)H₂** in C_6D_6 at 298 K.

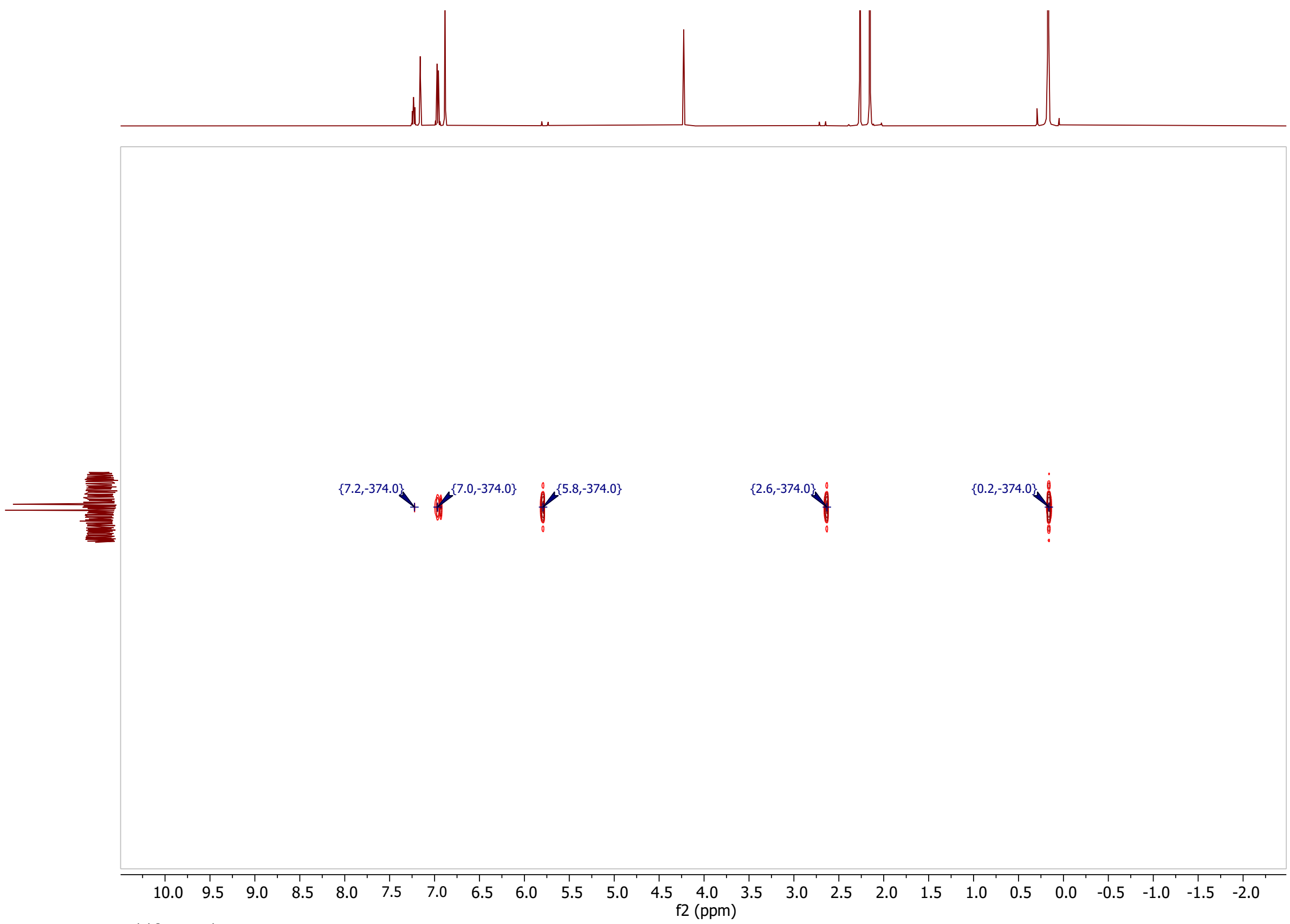

Figure S8. ^{119}Sn/^{1}H HSQC NMR spectrum of **(1)H_2** in C_6D_6 at 298 K.

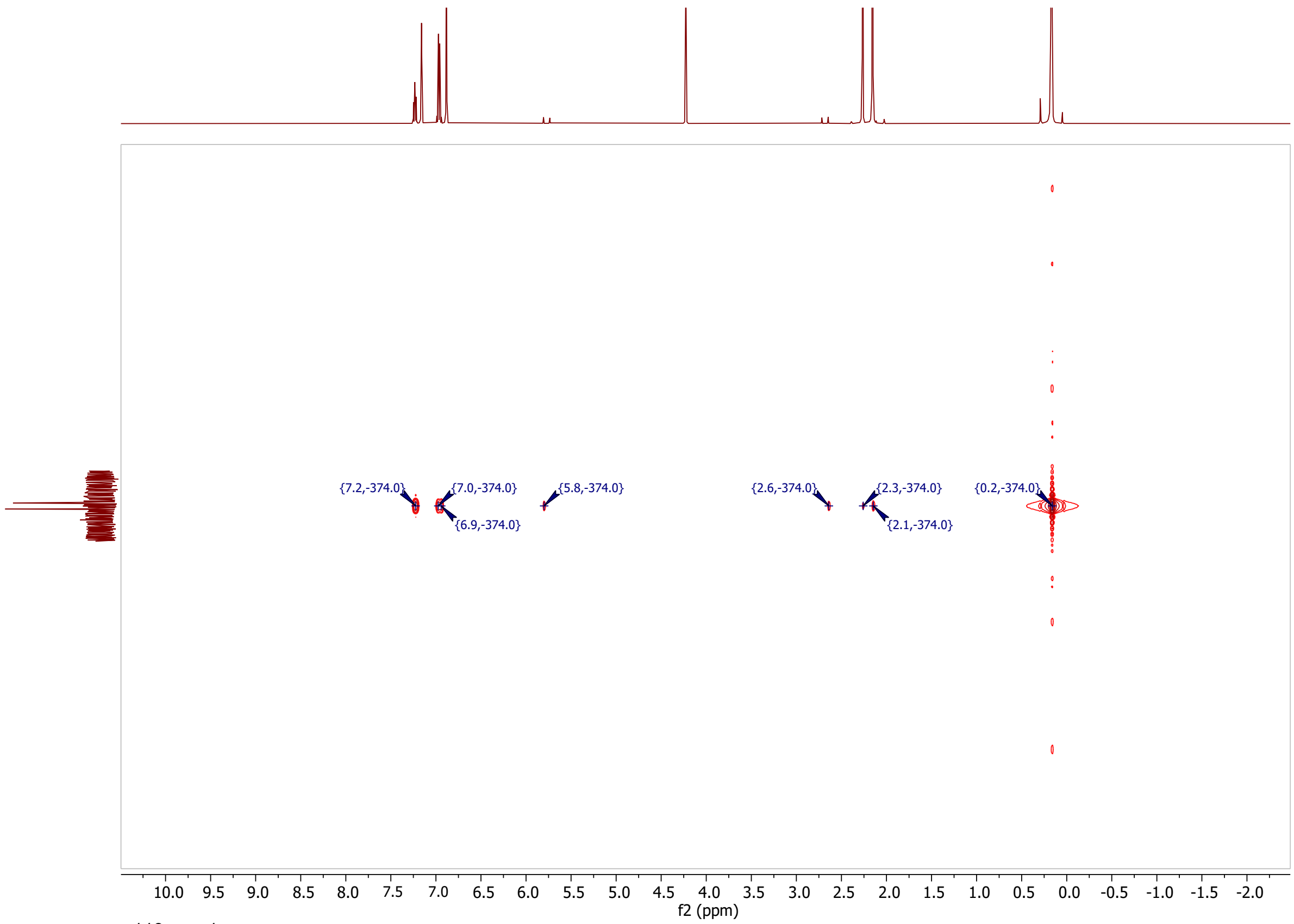

Figure S9. ^{119}Sn/^{1}H HMBC NMR spectrum of **(1)H₂** in C_6D_6 at 298 K.

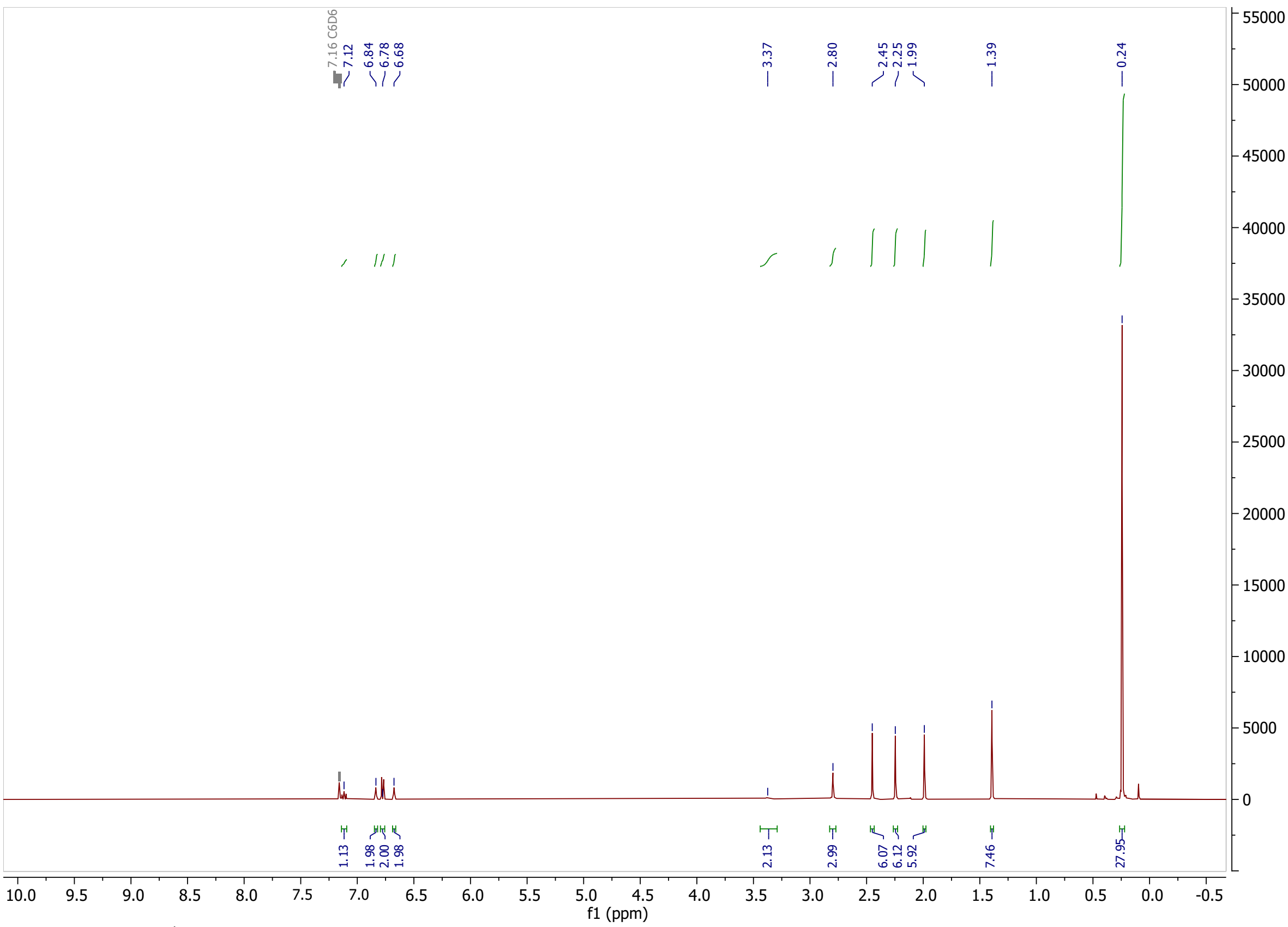

Figure S10. ^{1}H NMR spectrum of **1·IMe₄** in C_6D_6 at 298 K.

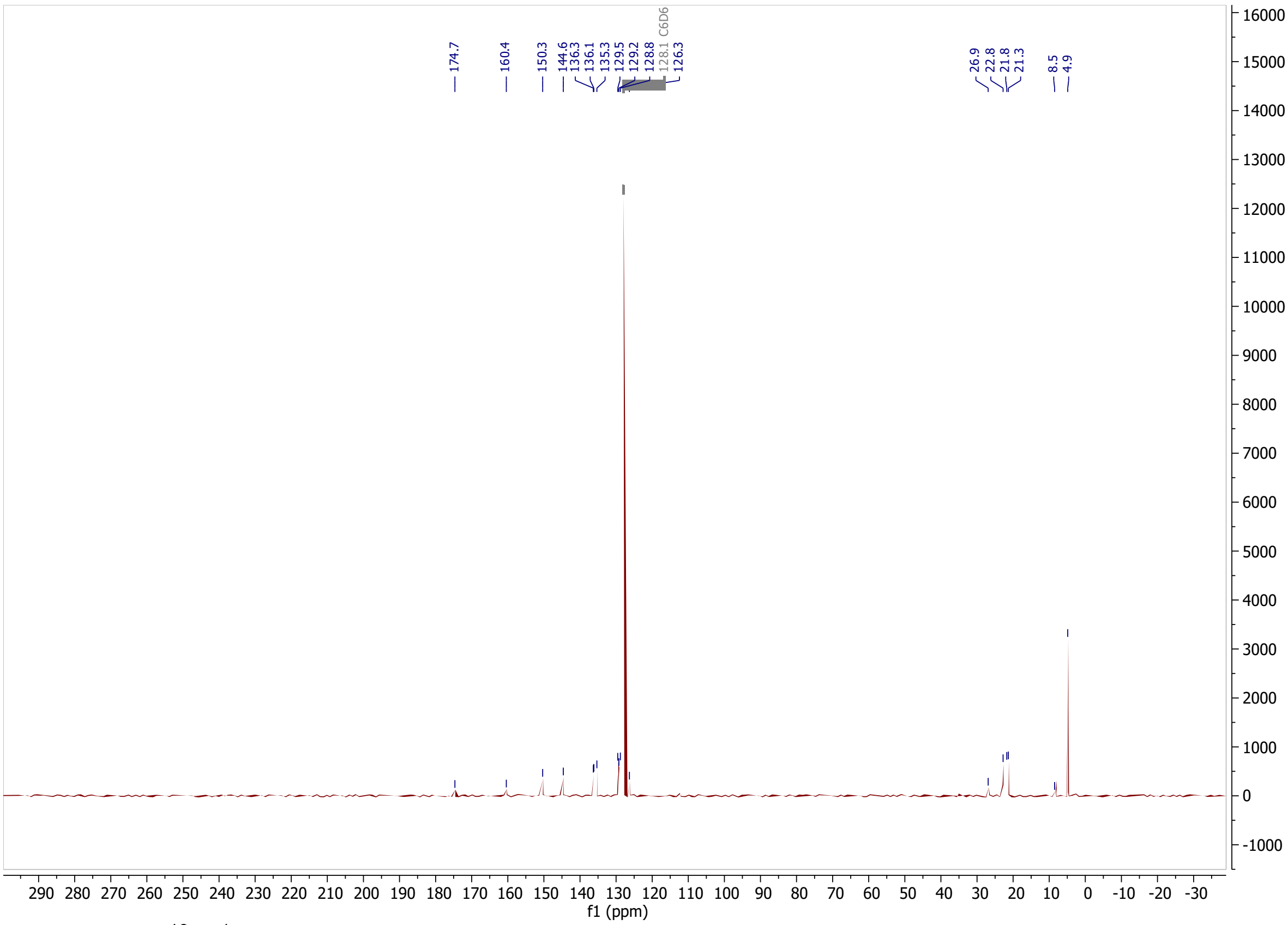

Figure S11. $^{13}C\{^{1}H\}$ NMR spectrum of **1·IMe4** in C_6D_6 at 298 K.

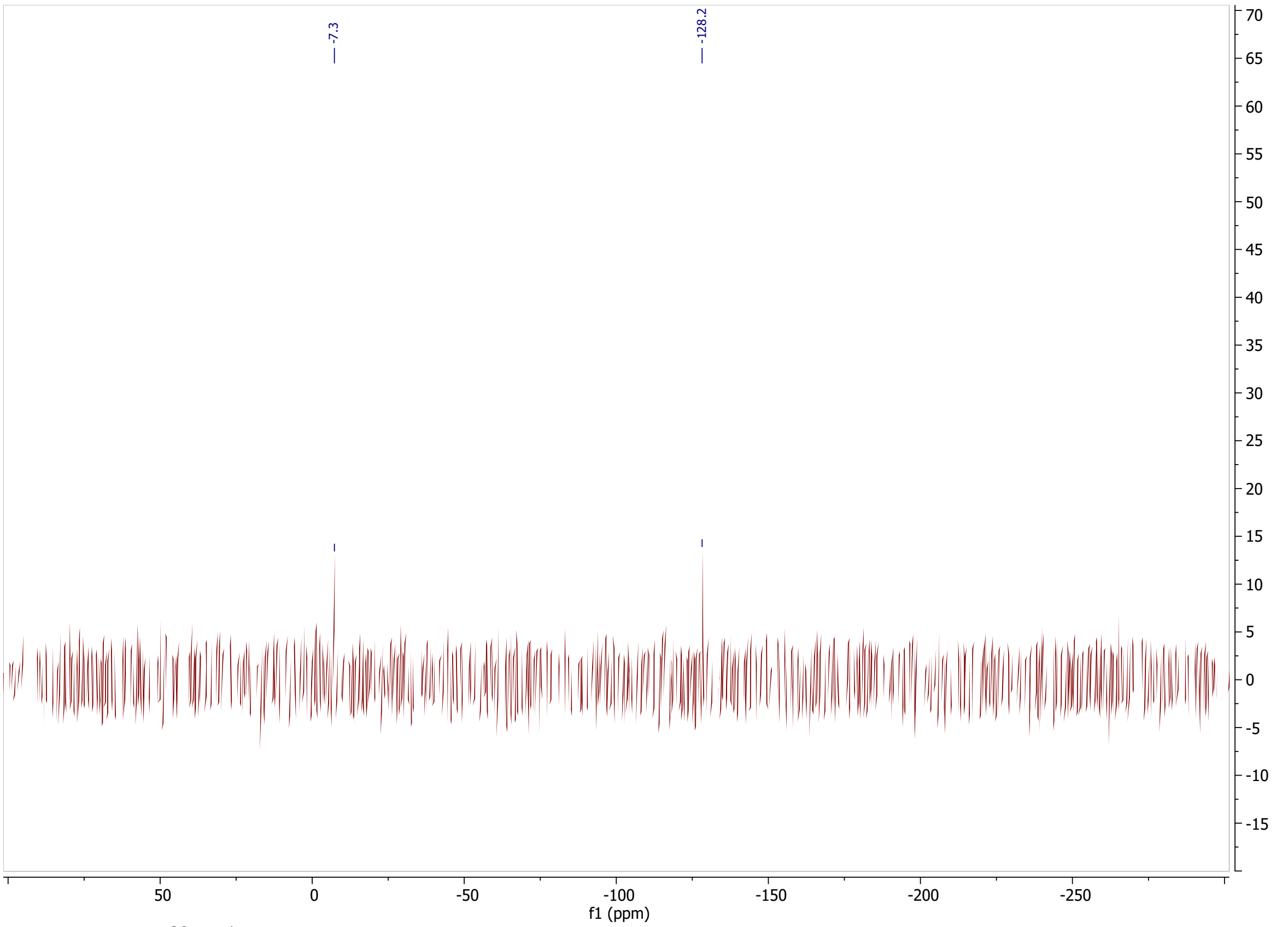

Figure S12. $^{29}Si\{^{1}H\}$ NMR spectrum of **1·IMe4** in C_6D_6 at 298 K.

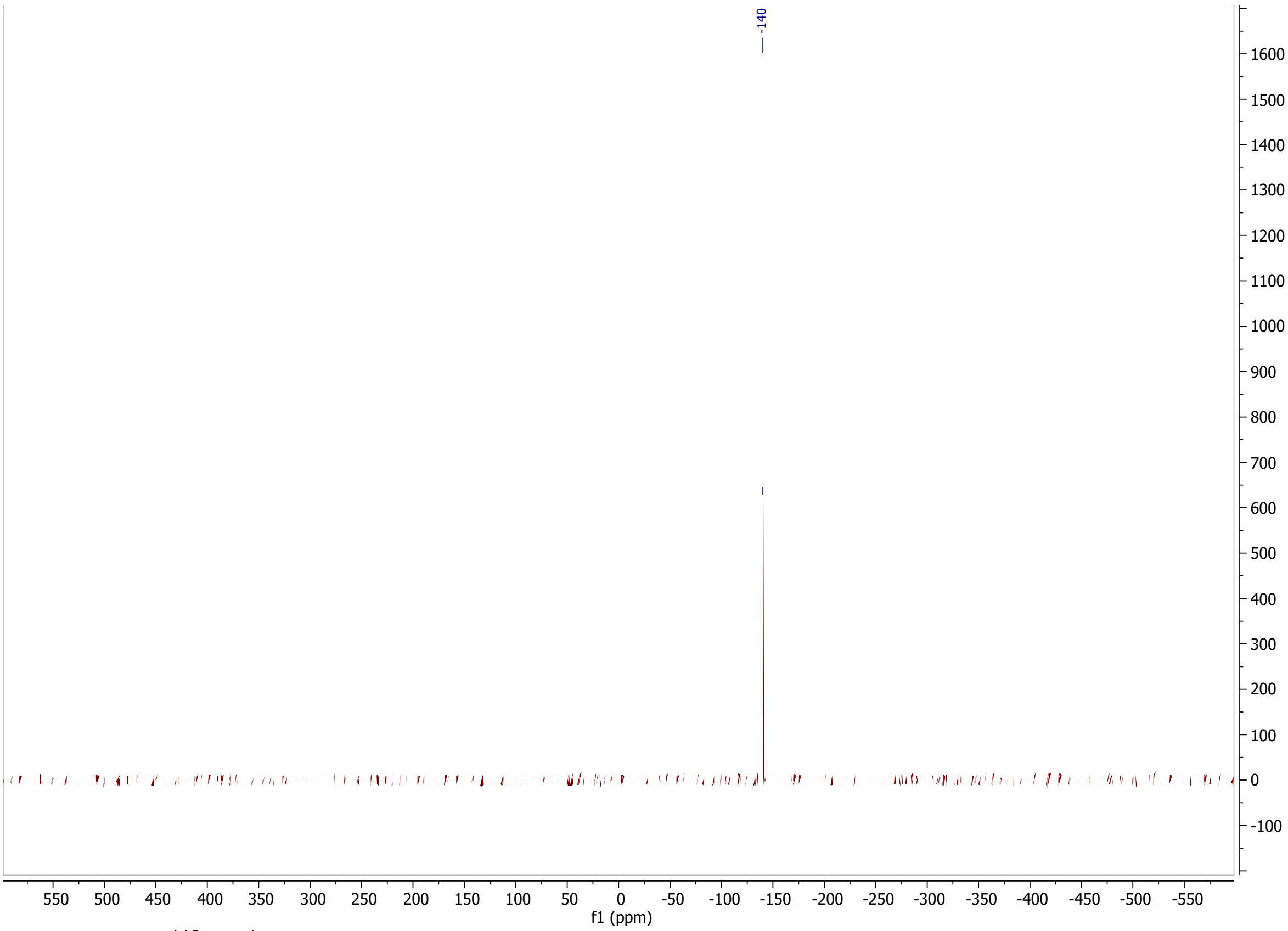

Figure S13. $^{119}Sn\{^{1}H\}$ NMR spectrum of **1·IMe₄** in C_6D_6 at 298 K.

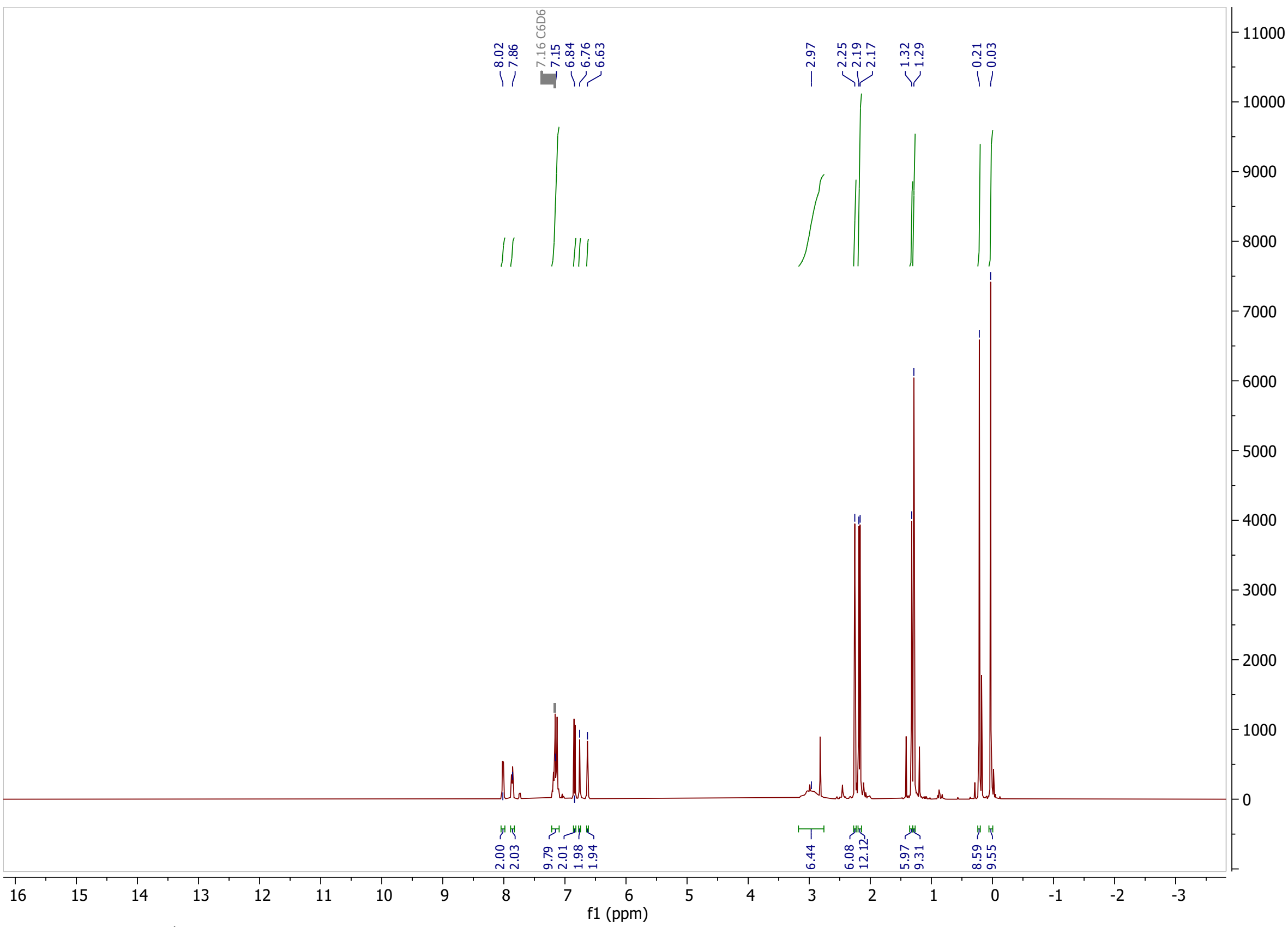

Figure S14. ^{1}H NMR spectrum of **2·IMe₄** in C_6D_6 at 298 K.

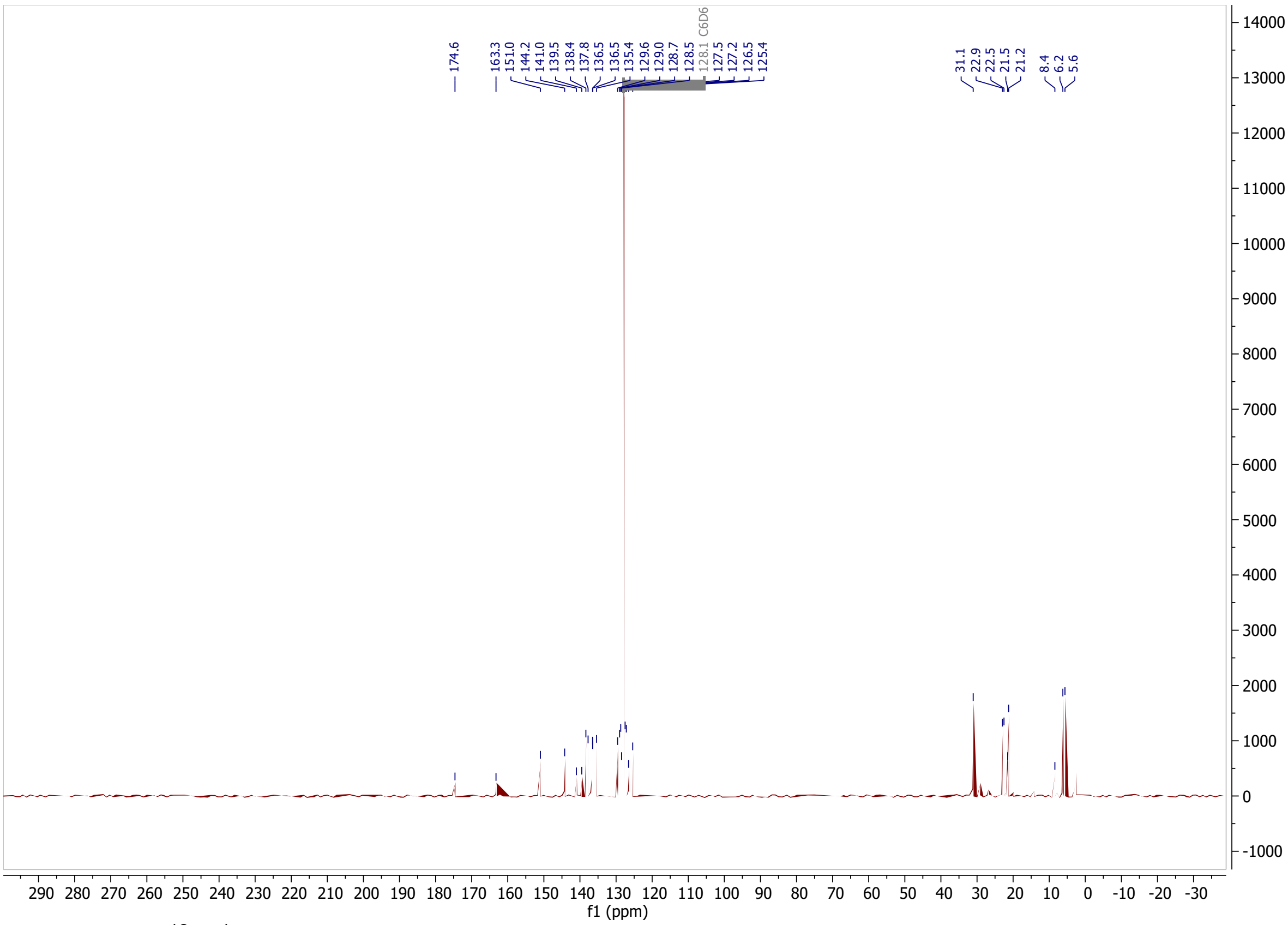

Figure S15. $^{13}C\{^1H\}$ NMR spectrum of **2·IMe₄** in C_6D_6 at 298 K.

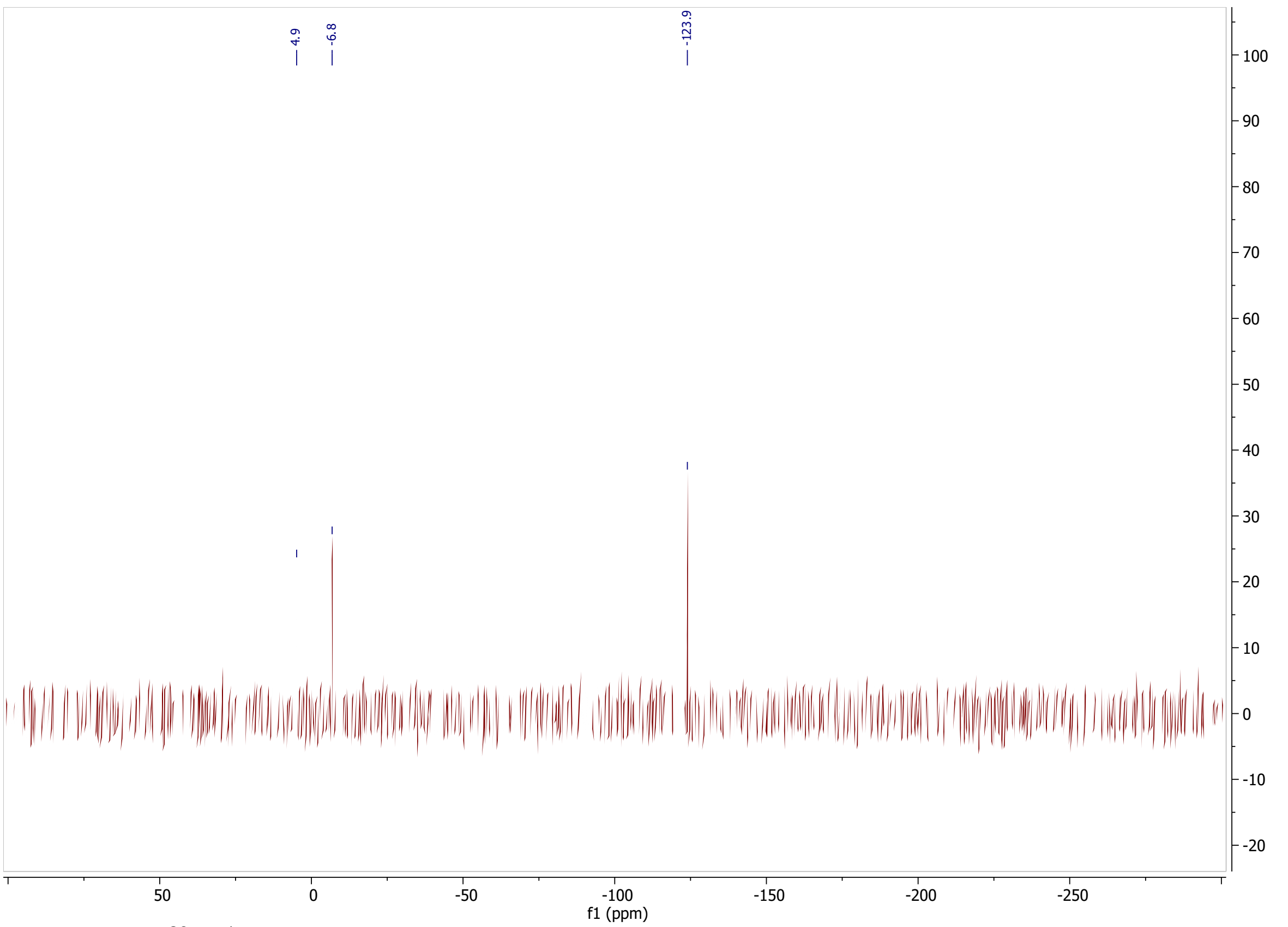

Figure S16. $^{29}Si\{^1H\}$ NMR spectrum of **2·IMe₄** in C_6D_6 at 298 K.

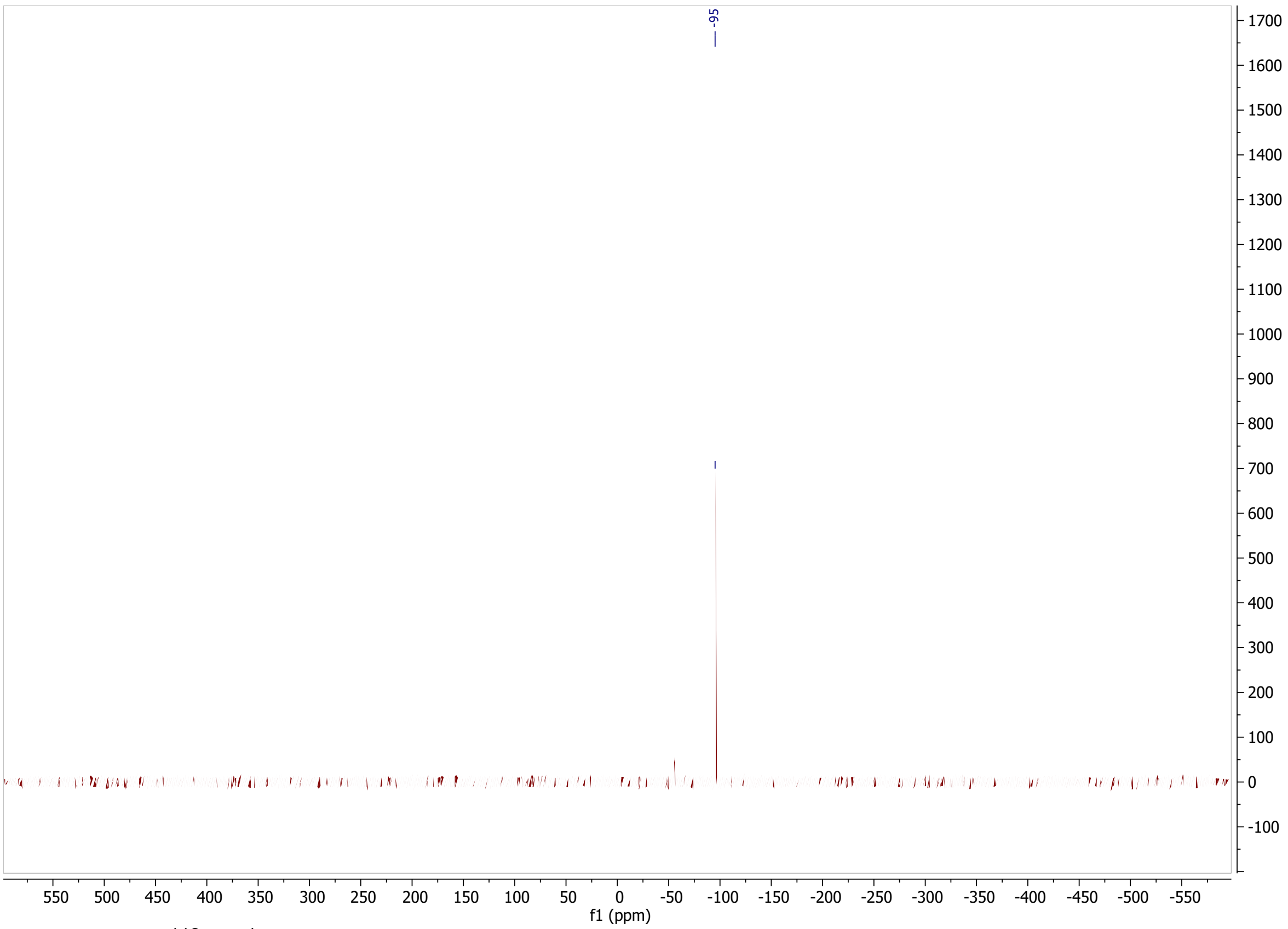

Figure S17. $^{119}Sn\{^1H\}$ NMR spectrum of **2·IMe₄** in C_6D_6 at 298 K.

6. Quantum Chemical Calculations

All computational work was performed using ORCA (revision 6.1.1). [s8,s9] The transition state associated with H_2 activation was investigated using the meta-generalized gradient approximation (mGGA) functional r²SCAN,[s10] in conjunction with the def2-TZVPPm basis set,[s11] including the D4 dispersion correction,[s12] and geometrical counterpoise correction (gCP),[s13,14] (collectively referred to as the r²SCAN-3c method).[s11] Solvent effects were approximated using the conductor-like continuum polarization model (CPCM) with benzene as the solvent.[s15]

The transition-state structure was characterized by vibrational frequency analysis and exhibited a single imaginary frequency of −1209.13 cm^{-1}. Inspection of the corresponding normal mode confirmed that it represents the expected reaction coordinate for H_2 activation.

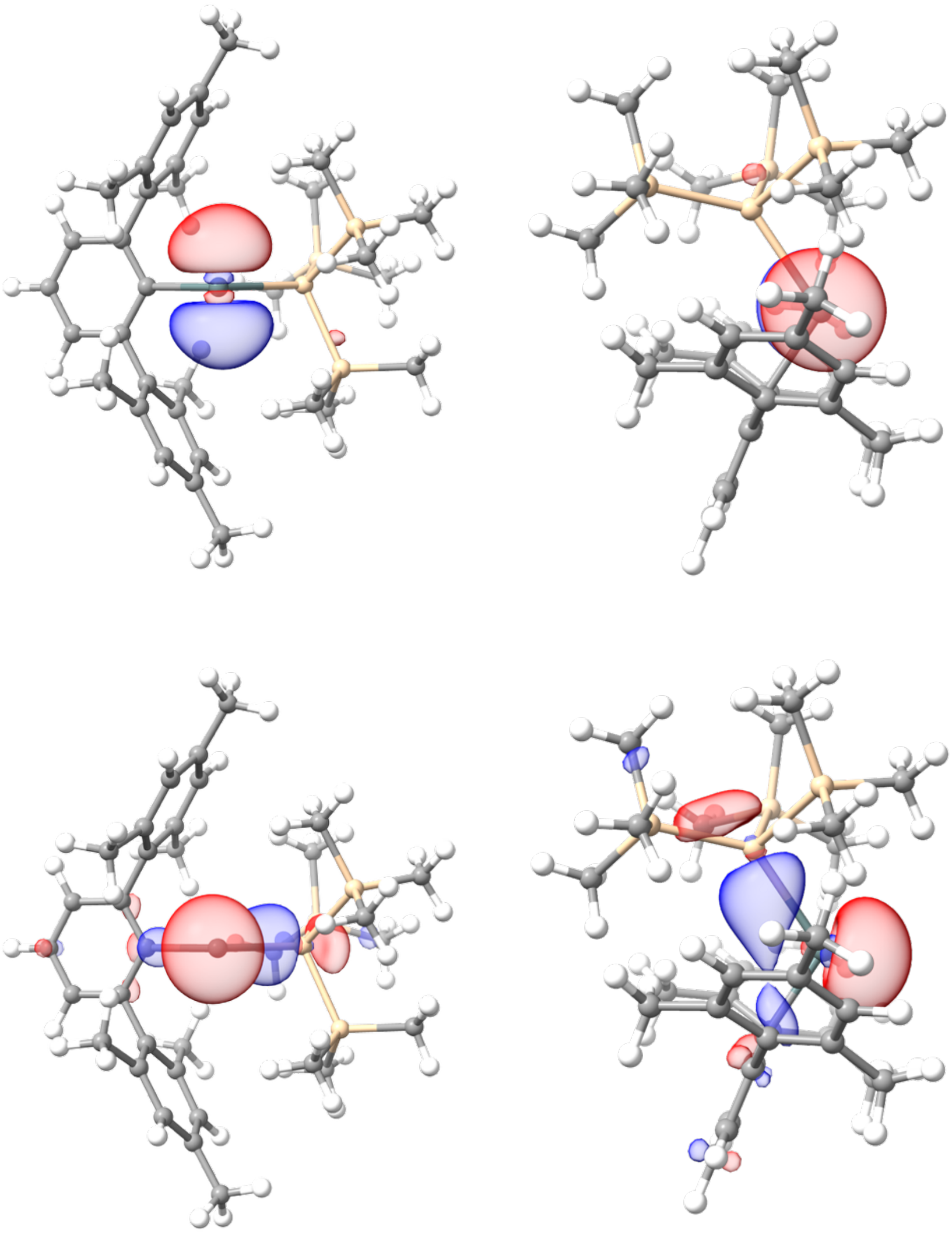

Figure S18. HOMO (lower) and LUMO (upper) for stannylene **1**. (isovalues 0.05; HOMO: orbital 164, -0.170003 a.u., -4.626016 eV; LUMO: orbital 165, -0.100093 a.u., -2.723657 eV)

XYZ Coordinates:

1: E -2660.946246025238

Sn	18.03028224622459	5.60473465872089	31.34126208783658
Si	18.12910249840785	8.09022140612828	32.27288136922957
Si	19.80664590800517	8.86256358697011	30.78559054080476
Si	18.58695950091792	9.03855134903984	34.38631862496775
Si	16.12859466984660	9.04845823674435	31.43626399307553
C	19.27269519641765	8.63117757775242	28.98464528497736
H	20.08524887936086	8.94214324418368	28.31558511657881
H	18.38652413247884	9.22501360278030	28.73747925053857
H	19.04258482830667	7.58109722508494	28.77195443911982
C	21.39521291549262	7.86721790856499	31.03383463396390
H	21.23865199828286	6.80138699819478	30.82572870208974
H	21.76103651893062	7.94447045115117	32.06355586779375
H	22.18343631505344	8.23005804844732	30.36148120212859
C	20.23661376704129	10.68836429544981	31.03660059294594
H	20.97915727637290	10.99270902344780	30.28760606151126
H	20.66806856587424	10.86830528487831	32.02701771157606
H	19.35981178502929	11.33445890674743	30.92630211421314
C	20.42737723373332	8.85435878357060	34.78164790668362
H	20.77571242651973	7.83012443396346	34.62206228786835
H	20.62790593495895	9.12988130702128	35.82464669025089
H	21.02259578533130	9.51183531622056	34.13804474325684
C	17.54939867462647	8.31024626179847	35.78734879090412
H	17.71823843494074	7.23814061515004	35.92698823348877
H	16.48102030069674	8.45933178396584	35.59472977042084
H	17.79647004533843	8.81751234779343	36.72878075571268
C	18.20880967502802	10.89376902123498	34.34228015321530
H	18.75432125266700	11.39766506668471	33.53804642591221
H	18.50800282887589	11.35120433331487	35.29449852792663
H	17.14058731099552	11.08645472393543	34.19871263639544
C	14.83753574873271	9.16814907289852	32.81151463343893
H	13.86407260611311	9.45070005171711	32.38989853764277
H	15.11830767216699	9.92577195035645	33.55116508398836
H	14.71745918434146	8.21410153487817	33.33097322468093
C	15.42497137551867	7.99220581938160	30.03513116581742
H	16.14643767301161	7.86940073141692	29.21973866714614
H	14.52336288619327	8.46293581723678	29.62160262208307
H	15.14434511427087	6.99410746619777	30.39443924303996
C	16.38818813639892	10.80433372939272	30.78094900975685
H	17.06084129257673	10.82963370420507	29.91769980109563
H	16.80495351410763	11.45965279199668	31.55346564857256
H	15.42224019970725	11.22235696979503	30.46834933700144
C	17.75447087770679	4.12535350875318	32.95776125920164
C	18.86243583143370	3.48304704821905	33.52581798043145
C	18.68277187521150	2.36883533011141	34.34875889758453
H	19.54988427938452	1.87966356097615	34.78823439823309
C	17.40184128204006	1.88169275170992	34.59698795311464
H	17.26490192408081	1.01320779155891	35.23556388205509
C	16.29793416685301	2.49721241605460	34.01224024271019
H	15.29630850852043	2.10955347707378	34.18799941614559
C	16.47238420529765	3.61032092988002	33.18620736365589
C	20.22851218386531	4.00005052375490	33.22002555320816
C	20.88829081820010	3.60691162209675	32.03933967614109
C	22.16158573690893	4.11732310594431	31.77264184226907
H	22.66902512390050	3.81478088890577	30.85832276571213
C	22.80176131219652	4.98615366887517	32.65350293914101
C	22.14592259815180	5.33679631993886	33.83485481403449
H	22.64189832461949	5.99825172458001	34.54325054006047
C	20.87119666908488	4.85838292135655	34.13195137321443
C	20.28821859547774	2.57514123472577	31.11991485527258
H	20.80719769827991	2.56106515188297	30.15800555130050
H	19.22343619830432	2.74989557972555	30.93942586140483
H	20.37186410936543	1.57637496255950	31.56677850982522
C	24.15196596302300	5.55974038633195	32.32422577163990
H	24.74577122388686	4.86412174710221	31.72385228188252
H	24.71250233078134	5.80608291649197	33.23076312912958
H	24.03979225734010	6.48416885752745	31.74342276760944
C	20.19482979566123	5.22417152969860	35.42451629783718
H	19.20923151951837	5.66344346544799	35.23539220337797
H	20.79255948404234	5.93585066326413	35.99923022677036
H	20.02753070904430	4.33612655241675	36.04428072885838
C	15.30350775414682	4.24921032009935	32.51176318880518
C	14.56607154434069	5.24670093996599	33.17790407478905
C	13.45051490059411	5.79989613242216	32.55170654199755
H	12.87364410165109	6.56120819048437	33.07331001415886
C	13.05783110977356	5.40489022453845	31.27215420510970
C	13.79840042278694	4.41691218860643	30.62629526982697
H	13.49968081949576	4.08961961723822	29.63215693290530
C	14.90852931015221	3.82077465664202	31.22904044286671
C	14.96963532782816	5.68848711459564	34.55740555437827
H	15.99021752935296	6.08879458240224	34.55264329028705
H	14.96591380103165	4.84750918880881	35.25975312092761
H	14.29561123062245	6.46004969853289	34.93878405935781
C	11.88722155301005	6.06109561620626	30.59372043129025
H	12.21195589192237	6.96243662357127	30.05835914204490
H	11.12787671274926	6.36719696226465	31.31944754416442
H	11.42347021301909	5.39307406767417	29.86223107858976
C	15.61964294126424	2.68787520890418	30.53795187549829
H	15.32741554520874	2.62934615549346	29.48635422117464
H	15.37137267455088	1.73154883153570	31.01499157645388
H	16.70762662810901	2.79447374142419	30.59290710615922

(1)H₂ E -2662.137892274126

Sn	18.06324652859031	5.73391080949387	31.51147971233646
Si	18.09311949014490	8.25298458799541	32.13740786048977
Si	19.81241559716684	8.96079392627299	30.66629218411937
Si	18.63401236823514	9.05294566458575	34.29476249244360
Si	16.03348170859730	9.20597411190738	31.45343011971504

C	19.28081664848576	8.61546510983933	28.88528407692437
H	20.07585802113513	8.91157205907241	28.18944074704852
H	18.37506917926401	9.17291690642655	28.62175886596423
H	19.07691722611468	7.54999247055738	28.72833391155617
C	21.43158908502292	8.04742276047678	31.00639008714468
H	21.31849232643609	6.96101875119261	30.93175572298650
H	21.81251338216219	8.27676806271788	32.00728772621847
H	22.18785181693114	8.36353308460895	30.27623451397186
C	20.17524117907647	10.80858498471613	30.84303313615676
H	20.90856581465042	11.10727262774980	30.08298235349058
H	20.60429292562272	11.03432278196394	31.82548813115121
H	19.28116754287204	11.42592208661340	30.71205852401156
C	20.47072213753334	8.79835666203477	34.65103697975738
H	20.79840645854313	7.78249674258025	34.41179099121671
H	20.68683953793010	8.99134259386587	35.70930804957982
H	21.07122447592734	9.49228604543693	34.05199935700190
C	17.59254487424881	8.33104972416264	35.69368569687587
H	17.74049869384130	7.25538552201927	35.82463676596220
H	16.52553302359485	8.50684536129657	35.51921015985363
H	17.86658628427242	8.82605420778723	36.63430090178640
C	18.31154631457886	10.91787073449903	34.28221190912765
H	18.85463639887056	11.41899100069408	33.47462726426473
H	18.64668507589001	11.34943191268677	35.23432677842542
H	17.24718415863844	11.14740685409412	34.16697998212864
C	14.83483199795565	9.29048842191971	32.91118161235484
H	13.85699506070522	9.65493352200303	32.57116989883893
H	15.19553361067010	9.96777835746699	33.69246474235164
H	14.68930722445445	8.30252870493996	33.35794580768864
C	15.21529546996160	8.22007591567120	30.06991522760994
H	15.87827054870004	8.10172929564617	29.20580804321209
H	14.31108727909753	8.74639046163682	29.73758017692057
H	14.92368080955475	7.22087296068959	30.41077344128290
C	16.32037284242569	10.96877188712595	30.83186516183939
H	16.93501577421239	10.97938523696076	29.92518333968771
H	16.81654149921972	11.58979398451330	31.58529769287237
H	15.35622718040659	11.43459688501644	30.59085194054054
C	17.77024372512974	4.04117563907655	32.85121604659986
C	18.87377726747438	3.38792528761680	33.43018810882463
C	18.68021423972695	2.23915015945605	34.20245329739484
H	19.54484134415958	1.74619992483833	34.64133372427449
C	17.40301414342985	1.73148792790148	34.40881205148269
H	17.26070191238642	0.83795086062176	35.01067151686094
C	16.30921178064841	2.37272405272121	33.84117428940419
H	15.30437577910411	1.98553759783199	33.99480298841182
C	16.48108562149122	3.52309969160483	33.06402827359406
C	20.26268219874170	3.90166583905821	33.24981047547147
C	21.03881068904062	3.47663029442880	32.15913560835976
C	22.32226611388883	3.99739628232083	31.99750873664787
H	22.91680850469193	3.67888629495202	31.14296074344105
C	22.85922469099583	4.91642977362219	32.89982177756706
C	22.08970381112850	5.29084373383184	34.00000797257627
H	22.50394243271868	5.98348580570334	34.73067049311252

C	20.79811899276822	4.79514743037368	34.19017356161126
C	20.49381715693005	2.47948809947149	31.17408013497758
H	21.21681231385564	2.28189912144026	30.37858019844857
H	19.56683911333996	2.84258329163863	30.71444428588378
H	20.25009711801987	1.53072641326173	31.66599452909671
C	24.22088179069795	5.51294883740941	32.67119114403371
H	24.89521982660099	4.79714790310671	32.19131224029494
H	24.67442701313935	5.84437045743115	33.60986069524941
H	24.14807385943796	6.38782119273386	32.01232386716999
C	19.99912273895233	5.18861438870927	35.40289027563053
H	19.00183942146575	5.54188364667032	35.11919166986518
H	20.50127633477102	5.97508864411199	35.97180384741694
H	19.84693748272480	4.32918023078271	36.06658191730243
C	15.26840105771211	4.16202473027523	32.47445134638125
C	14.61422617877653	5.19528284904986	33.16207913176470
C	13.46279845222371	5.75884221778918	32.60723746320316
H	12.94833464115421	6.55090870903572	33.14816861041246
C	12.95818267789894	5.33132965532750	31.38112740293329
C	13.62103781929764	4.29902827339524	30.71560030161436
H	13.23776730926556	3.94884203046137	29.75868840376519
C	14.76285432751598	3.70122799900609	31.24681132218506
C	15.14596344089483	5.69506055397602	34.47769845843835
H	16.09146147475675	6.23349074478466	34.33579646916957
H	15.35481666223014	4.87012982036583	35.16697248683486
H	14.43825266216704	6.37878576822458	34.95451159428323
C	11.75039960568750	5.98843758118705	30.77085777818768
H	12.05415666793137	6.75168650692280	30.04302200789893
H	11.13912417926601	6.48237657607809	31.53159474516026
H	11.12687778490900	5.26236207299443	30.24006095067332
C	15.45174735102675	2.58865685278898	30.50529523969891
H	14.97142705010795	2.40585062728790	29.54065987970642
H	15.43578362023140	1.65636928999649	31.08150094584965
H	16.50670360548043	2.82946420434179	30.32740001520678
H	16.88464427399738	5.54174899949909	30.27416965006192
H	19.54366895608438	5.42144878013922	30.69511118279759

TS: E -2662.070534072802

Sn	18.00053238409462	5.75503934579198	31.57741247080868
Si	18.20999850891563	8.33125537278949	32.01645139800778
Si	20.00389749606658	8.97278029273840	30.62466110709508
Si	18.64269978729993	9.02595802838342	34.22768267616510
Si	16.18200442009483	9.28905222184346	31.26848538493648
C	19.49477327993367	8.79193116341302	28.81455239150118
H	20.34443705151083	9.00954301019311	28.15537060457210
H	18.68228743234065	9.48099010183333	28.55837809513411
H	19.15272556832210	7.77253365897948	28.60168863508056
C	21.51150339196095	7.88435385409558	30.94091417325206
H	21.29229901507845	6.83038690884472	30.73487910343566
H	21.84695543158468	7.95148312430732	31.98156064427662
H	22.34199328192378	8.19194255225296	30.29267434073640
C	20.47753673680449	10.77679973940565	30.94203486581807

H	21.26286702419980	11.08572744193995	30.24065962358976
H	20.86051826520053	10.91831562140137	31.95854491260280
H	19.62188497960918	11.44718624330047	30.80724669071552
C	20.46172785692097	8.74551409726586	34.64750403813760
H	20.77799166200661	7.72516983815968	34.40980252491196
H	20.64190220646670	8.92308176435534	35.71518181690940
H	21.09804357537394	9.43266548015504	34.07839454804850
C	17.54291273971324	8.16020860861674	35.49008783832389
H	17.69995955233626	7.07762932039253	35.49081702441367
H	16.48416924616900	8.34428443157273	35.27829616281895
H	17.76054491137300	8.53632935661232	36.49774911176127
C	18.29708350874983	10.88249014007581	34.33568578719660
H	18.86820890837667	11.44616622536880	33.59027574580350
H	18.58061027102235	11.25172462668375	35.32965223792344
H	17.23441525856068	11.10105537299553	34.18551804749374
C	14.87128314479356	9.14421292133502	32.61999908469762
H	13.90082625627130	9.49159320504177	32.24309565430148
H	15.12814631467115	9.74297975937544	33.50056920677962
H	14.75814945971532	8.10031528800871	32.93356684495946
C	15.51421021840970	8.48137592539136	29.69731741312574
H	16.27156046437388	8.42922987538696	28.90802268097881
H	14.67374689509817	9.07895587876043	29.32137984692109
H	15.14664726086127	7.46768027155982	29.88718245868898
C	16.47565015188668	11.11757731531276	30.87851688653412
H	17.15415088000962	11.23289966018258	30.02570405664544
H	16.90608155101388	11.65757321160946	31.72812055275501
H	15.52465567087152	11.59903660467977	30.61689672456966
C	17.71225100794757	4.10540732565533	33.00830476436506
C	18.86151215697153	3.52778404226246	33.57520494875076
C	18.74784183168850	2.41373692725531	34.41379222142371
H	19.64920638438305	1.98215687837047	34.84316228364393
C	17.50515867461012	1.85591987021956	34.68352499879662
H	17.42351977720460	0.98884549915895	35.33332473252054
C	16.36775401690142	2.40353975491177	34.10138617122725
H	15.39044700485208	1.96373121875016	34.28775114299253
C	16.45643399707771	3.51719834816802	33.26101504617776
C	20.22610151857852	4.05614957672675	33.28360972096704
C	20.87999792400128	3.68854202747162	32.09122250502663
C	22.16344734861226	4.17537185170166	31.84480809278608
H	22.66695286338146	3.89086394117207	30.92244907725938
C	22.82123026619330	5.00477848416608	32.75359753963843
C	22.17391243574515	5.32307974873404	33.94590197862698
H	22.68279589174617	5.94621569468925	34.67948268596280
C	20.89023074412677	4.85311982082065	34.23061756982380
C	20.23293506548448	2.74510365108195	31.11261125641622
H	20.93454912271783	2.46738773192751	30.32198428213668
H	19.35032381155533	3.19297968181480	30.63872924206508
H	19.89028858030322	1.83357861920569	31.61474982637544
C	24.18447866362311	5.55639957081287	32.44035712386235
H	24.79891190207919	4.82201147436292	31.91021515883709
H	24.71098245147934	5.85994805837091	33.34971368026015
H	24.09944402135956	6.43923104485225	31.79412812741262
C	20.25132367858551	5.16013529509504	35.55811554802305
H	19.20034270199566	5.43972077393909	35.44126351171637
H	20.77695663839616	5.96859009988704	36.07283410206355
H	20.26839646333028	4.27852821798981	36.21037834720612
C	15.20908343875545	4.03074634607081	32.62667406154185
C	14.43636642861574	5.00444825473626	33.28015068248327
C	13.32194371436701	5.53617435450688	32.63234806570062
H	12.73408069789255	6.30307060813620	33.13430559397939
C	12.95619729039947	5.12285813954837	31.35084331615536
C	13.71944942044932	4.13398448573258	30.73203995384702
H	13.44525724890613	3.79441480229442	29.73487100904872
C	14.83726047144757	3.57298391430173	31.35440073740744
C	14.83020657395899	5.49449172980473	34.64480483858360
H	15.85851971931292	5.87204365211146	34.63485992772261
H	14.79868075414341	4.68525998173090	35.38352113635689
H	14.16937677570675	6.29756186587454	34.98230753409061
C	11.75344901915580	5.71693708303131	30.66881435470810
H	11.69153310696847	6.79534091062736	30.84767149271420
H	10.82684510526658	5.26987302218817	31.04962813371030
H	11.78488345834491	5.54678218577939	29.58895337678480
C	15.63681788712946	2.50678639515240	30.65395791151206
H	15.24886500979172	2.32705936218252	29.64796428436004
H	15.60536823601186	1.56184710150141	31.20914408000039
H	16.69289071893423	2.78865265001510	30.57215196768436
H	16.20334379009021	6.28224621483330	31.74809824252398
H	16.53347695722564	5.65846129643733	30.59513214438559

H_2: E -1.169549800190

H	3.89139363129019	1.04292100000000	0.01365300000000
H	3.14946636870981	1.04292100000000	0.01365300000000

7. References

s1 N. Kuhn and T. Kratz, *Synthesis (Stuttg).*, 1993, 561–562.

s2 M. Fischer, M. M. D. Roy, L. L. Wales, M. A. Ellwanger, C. McManus, A. F. Roper, A. Heilmann and S. Aldridge, *Angew. Chem. Int. Ed.*, 2022, **61**, e202211616.

s3 A. J. Murray, L. L. Wales, M. Dietz, E. M. Poland, C. McManus, A. E. Crumpton, J. J. C. Struijs and S. Aldridge, *Organometallics*, DOI:10.1021/acs.organomet.6c00001.

s4 B. J. Cosier and A. M. Glazer, *J. Appl. Cryst.*, 1986, **19**, 105–107.

s5 G. M. Sheldrick, *Acta Crystallogr., Sect. A:Found. Adv.*, 2015, **71**, 3–8.

s6 G. M. Sheldrick, *Acta Crystallogr., Sect. C:Struct. Chem.*, 2015, **71**, 3–8.

s7 O. V. Dolomanov, L. J. Bourhis, R. J. Gildea, J. A. K. Howard and H. Puschmann, *J. Appl. Cryst.*, 2009, **42**, 339–341.

s8 F. Neese, *Wiley Interdiscip. Rev.:Comput. Mol. Sci.*, 2012, **2**, 73–78.

s9 F. Neese, *Wiley Interdiscip. Rev. Comput. Mol. Sci.*, 2025, **15**, e70019.

s10 J. W. Furness, A. D. Kaplan, J. Ning, J. P. Perdew and J. Sun, *J. Phys. Chem. Lett.*, 2020, **11**, 8208–8215.

s11 S. Grimme, A. Hansen, S. Ehlert and J. M. Mewes, *J. Chem. Phys.*, 2021, **154**, 064103.

s12 E. Caldeweyher, S. Ehlert, A. Hansen, H. Neugebauer, S. Spicher, C. Bannwarth and S. Grimme, *J. Chem. Phys.*, 2019, **150**, 154122.

s13 J. G. Brandenburg, M. Alessio, B. Civalleri, M. F. Peintinger, T. Bredow and S. Grimme, *J. Phys. Chem. A*, 2013, **117**, 9282–9292.

s14 H. Kruse and S. Grimme, *J. Chem. Phys.*, 2012, **136**, 154101.

s15 V. Barone and M. Cossi, *J. Phys. Chem. A*, 1998, **102**, 1995–2001.
